# Comparative effectiveness of semaglutide *versus* liraglutide, dulaglutide or tirzepatide: a systematic review and meta-analysis

**DOI:** 10.3389/fphar.2025.1438318

**Published:** 2025-05-15

**Authors:** Mohammad Amin Karimi, Mohammad Sadra Gholami Chahkand, Parisa Alsadat Dadkhah, Farzad Sheikhzadeh, Shayan Yaghoubi, Fatemeh Esmaeilpour Moallem, Mitra Sadat Deyhimi, Melika Arab Bafrani, Mehregan Shahrokhi, Amir Nasrollahizadeh

**Affiliations:** ^1^ School of Medicine, Shahid Beheshti University of Medical Sciences, Tehran, Iran; ^2^ Student Research Committee, Golestan University of Medical Sciences, Gorgan, Iran; ^3^ School of Medicine, Isfahan University of Medical Sciences, Isfahan, Iran; ^4^ School of Medicine, Iran University of Medical Sciences, Tehran, Iran; ^5^ Student Research Committee, Faculty of Medicine, Islamic Azad University of Ardabil, Ardabil, Iran; ^6^ School of Medicine, Tehran University of Medical Sciences (TUMS), Tehran, Iran; ^7^ School of Medicine, Shiraz University of Medical Sciences, Shiraz, Iran; ^8^ Tehran Heart Center, Cardiovascular Diseases Research Institute, Tehran University of Medical Sciences, Tehran, Iran

**Keywords:** semaglutide, tirzepatide, dulaglutide, liraglutide, GLP-1 (Glucagon-Like Peptide 1), HbA1c, type 2 diabetes mellitus, randomized controlled trials

## Abstract

**Background:**

This study seeks to compare the effectiveness of Semaglutide compared to Liraglutide, Dulaglutide, or Tirzepatide. Additionally, it aims to investigate the implications of transitioning from Dulaglutide or Liraglutide to Semaglutide.

**Methods:**

We searched PubMed, Scopus, Cochrane Library, Google Scholar, and Web of Science (ClinicalTrials.gov for unpublished records) from their inception to 5 February 2025, including observational cohort studies and randomized controlled trials. Analyses were conducted using Review Manager (RevMan) version 5.4.1 and STATA 17.

**Results:**

The meta-analysis comprised 16 studies and 5,997 patients. Semaglutide significantly reduced hemoglobin A1c (HbA1c) levels compared to Liraglutide (0.56; 95% CI: 0.19–0.94; p < 0.001). However, no significant differences were observed between Semaglutide and Liraglutide in terms of fasting blood sugar (FBS), body mass index (BMI), and weight change. In comparison to Dulaglutide, Semaglutide displayed superior efficacy in reducing HbA1c levels (3.72; 95% CI: 0.02–7.41; p = 0.05) and FBS (2.66; 95% CI: 0.26–5.07; p = 0.03). However, no significant differences were found in weight and BMI change. Tirzepatide exhibited a notable advantage over Semaglutide in reducing HbA1c levels (−0.45; 95% CI: −0.88 to −0.02; p = 0.04). However, no clear superiority was observed for weight and FBS change. Transitions from Liraglutide to Semaglutide did not significantly impact HbA1c levels. However, weight loss (2.48; 95% CI: 0.45–4.51; p = 0.02) and reduced FBS levels (10.76; 95% CI: 0.55–20; p = 0.04) were observed. Transitioning from Dulaglutide to Semaglutide did not significantly affect HbA1c levels and weight change.

**Conclusion:**

While the precise source of heterogeneity remains elusive across most studies, analyses consistently demonstrate Semaglutide’s superior efficacy compared to Liraglutide in reducing both HbA1c levels and weight. Moreover, it presents advantages over Dulaglutide, specifically in lowering FBS levels. However, Tirzepatide surpasses Semaglutide in its efficacy for reducing HbA1c levels.

## Graphical Abstract

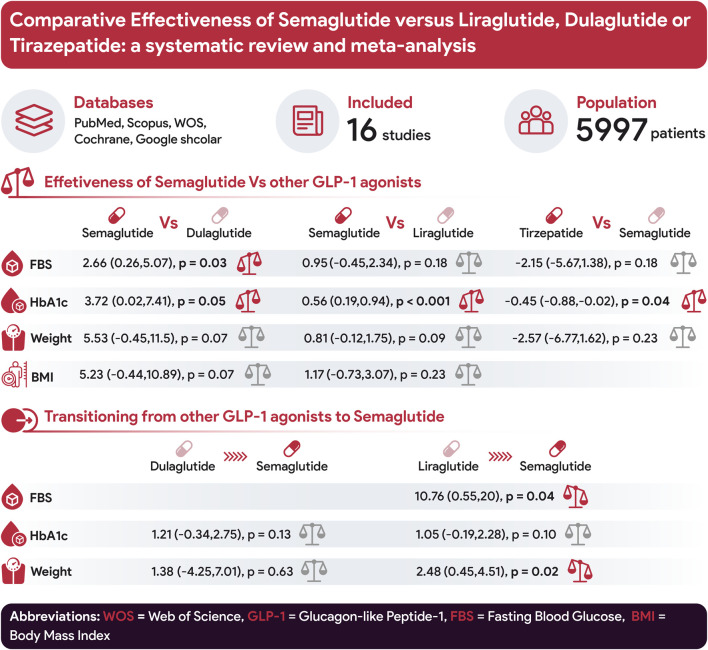

## 1 Introduction

An important global public health challenge is the increasing prevalence of metabolic syndrome, obesity, and diabetes mellitus, which pose a significant disease burden around the world (Garvey et al., 2016; Yumuk et al., 2015; Wharton et al., 2020). Significant weight reduction, blood sugar control, and lifestyle modification have improved health outcomes and quality of life (Mertens and Van Gaal, 2000; Li et al., 2014; Mokgalaboni and Phoswa, 2023). Various therapies, such as herbal regimens, are available for this issue, which demonstrate glycemic control limitations (Mokgalaboni and Phoswa, 2023). The study by Mokgalaboni et al. indicates that okra may function as an adjunct dietary nutrient, especially for pre-diabetic and Type 2 Diabetes patients, due to its potential to regulate hyperglycemia (Mokgalaboni et al., 2023). Amongst modern therapy options for these goals, glucagon-like peptide-1 (GLP-1) receptor agonists represent one of the most efficacious treatment classes, offering clinically important reductions in blood sugar level and body weight alongside a low risk of hypoglycemia (Buse et al., 2020; Shyangdan et al., 2011). The group of medications known as GLP-1, including exenatide, Lixisenatide, Liraglutide, Albiglutide, Dulaglutide, and Semaglutide, have emerged as effective therapeutic options for both conditions for the management of Type 2 diabetes mellitus (T2DM) and obesity (Wilding et al., 2021; Alfaris et al., 2024).

In patients with contraindications or intolerance to metformin and those with atherosclerosis, heart failure, or chronic kidney disease, it is suggested to add an analog of GLP-1 to metformin therapy (Hunt et al., 2019; Burcelin and Gourdy, 2017; Gourgari et al., 2017; Quattrocchi et al., 2020). Additionally, Semaglutide and high-dose Liraglutide are FDA-approved for obesity and may be prescribed to overweight patients who have comorbidities (Elliott and Chan, 2021; Nuffer and Trujillo, 2015). The utilization of GLP-1 analogs is a subject of ongoing research, showing promising outcomes in terms of HbA1c reduction and weight loss in patients with type-1 diabetes mellitus. However, notable challenges are associated with prescribing these medications, including their higher costs and potential tolerability issues (Care, 2019; Janzen et al., 2016; Hinnen, 2017).

Following oral sugar administration, GLP stimulates insulin secretion through the incretin effect (Vilsbøll et al., 2012). In patients with T2DM, this process can be interrupted or halted altogether. However, pharmacologically elevated levels of GLP-1 may help reinstate insulin secretion. One benefit of this treatment approach is delayed gastric emptying and the suppression of glucagon production from pancreatic alpha cells in response to increased blood sugar levels. Additionally, GLP-1 receptor agonists have been shown to reduce pancreatic beta-cell apoptosis and stimulate their proliferation (Garber, 2011; Gallwitz, 2011; Zheng et al., 2018).

Expanding upon these metabolic advantages, GLP-1 receptor agonists also demonstrate significant effects extending beyond glucose regulation. In addition to reducing blood pressure and overall cholesterol levels, this class of medications has also been noted to lead to an average weight loss of 2.9 kg compared to a placebo. Regarding cardiovascular effects, GLP-1 agonists may influence various aspects such as left ventricular ejection fraction, myocardial contractility, coronary blood flow, cardiac output, and endothelial function. They also demonstrate potential in reducing infarction size and lowering the overall risk of cardiovascular events. The additional function of GLP-1 is to increase sugar uptake in the muscles, decrease sugar production in the liver, neuroprotection, and more satiety due to indirect action on the hypothalamus. Among GLP-1 analogs, Semaglutide stands out as a once-weekly injectable option that has attracted significant attention owing to its robust sugar-lowering effects, weight loss benefits, and ability to reduce cardiovascular risk. Semaglutide, Dulaglutide, and Liraglutide operate through similar mechanisms by primarily enhancing insulin secretion and suppressing glucagon release (Monami et al., 2009).

Although several meta-analyses have examined the efficacy of GLP-1 receptor agonists in type 2 diabetes mellitus, most have included placebo-controlled trials or combination therapies, limiting direct comparisons between these agents. There remains a need for analyses that specifically compare GLP-1 receptor agonists as monotherapies, eliminating confounding factors such as co-administration of insulin or metformin to better assess their relative efficacy.

Our meta-analysis focuses on Semaglutide because it is one of the most potent GLP-1 receptor agonists, with compelling evidence for superior glycemic control and weight reduction. While previous studies have extensively compared Liraglutide and Dulaglutide (Chang et al., 2020; Taheri et al., 2019), the role of Semaglutide—especially in switching from other GLP-1RAs—remains underexplored. This study aims to systematically compare the efficacy of Semaglutide with Liraglutide, Dulaglutide, and the novel drug, Tirzepatide, in the treatment of obesity and diabetes. Specifically, it evaluates their effects on key clinical outcomes, including HbA1c reduction, FBS levels, weight loss, and body mass index (BMI). Furthermore, the study investigates the clinical impact of transitioning from Liraglutide or Dulaglutide to Semaglutide. Through a meta-analysis of randomized controlled trials (RCTs), this research provides evidence-based insights into the comparative therapeutic benefits of these GLP-1 receptor agonists, with a particular focus on Semaglutide’s role in optimizing diabetes and obesity management.

## 2 Methods

**FIGURE 1:**
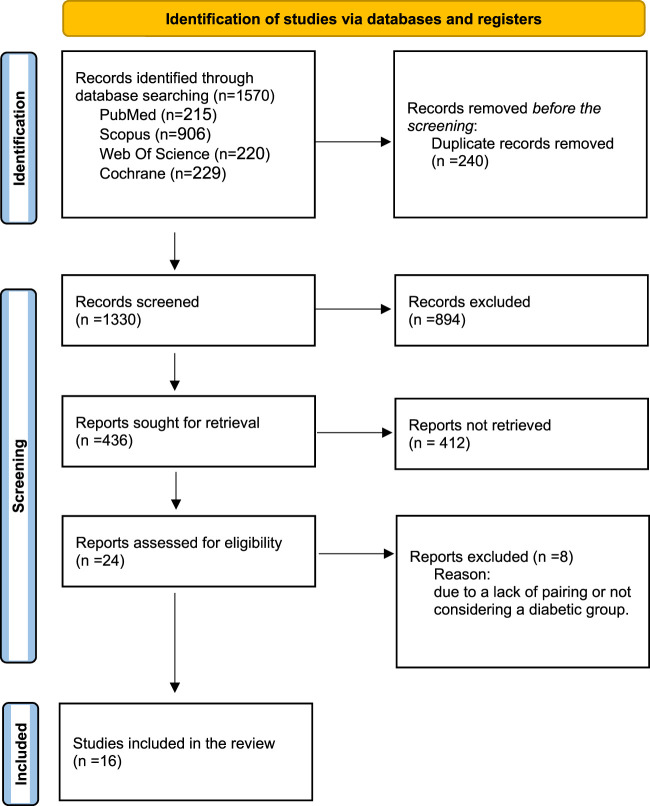
PRISMA flowchart illustrating the selection process of studies included in the systematic review and meta-analysis.

The present study adhered to the principles outlined in the Preferred Reporting Items for Systematic Reviews and Meta-Analysis (PRISMA; Figure 1) to conduct a comprehensive systematic review and meta-analysis (Page et al., 2021a). The study protocol was registered in the Open Science Framework (OSF), the International Prospective Register of Systematic Reviews. The provided link directs to the OSF database, which contains a record with the identification (https://osf.io/u2axy).

### 2.1 Literature search

#### 2.1.1 Search strategy

The initial step involved the identification of relevant keywords utilizing Medical Subject Headings (MeSH) terminology. Subsequently, a thorough search was conducted up to February 2025. The investigation encompassed databases, including PubMed, Scopus, Cochrane Library, and Web of Science, along with Google Scholar as a search engine. Furthermore, an investigation was conducted on databases such as ClinicalTrials.gov to find unpublished records. To enhance the specificity of the search, all searches were performed using the primary keywords, including Semaglutide and Tirzepatide (also their trade names). The search strategy was enforced by considering the title, abstract, and appropriate keywords and tags, utilizing sophisticated search functionalities for each search engine. No specific restrictions concerning time and language were imposed. The overview of our search strategy can be seen in Supplementary Table S1. Following a comprehensive search, elimination of duplicate entries, and retrieval of relevant publications, two researchers thoroughly examined the titles and abstracts of the selected studies. In cases where discrepancies arose, a third member was consulted to facilitate resolution through discussion. Subsequently, the subsequent stage entailed a comprehensive examination of the full text of the discovered articles to ascertain their precise adherence to the inclusion criteria. All studies that satisfied the predetermined inclusion criteria were incorporated into the analysis.

Our study exclusively incorporated observational cohort studies, encompassing retrospective and prospective designs and randomized controlled trials. Additionally, we considered early-phase clinical trials published in peer-reviewed publications and possessed comprehensive analyses. The primary objective of our investigation was to analyze scholarly research that investigated the effects of Semaglutide and Tirzepatide on individuals with diabetes and their impact on weight reduction. After employing Endnote version X20, duplicate studies were deleted, and relevant publications were retrieved. Subsequently, two researchers independently assessed the titles and abstracts of the identified studies, with any discrepancies being resolved by discussion, including a third member. Later, the subsequent stage entailed a comprehensive examination of the full text of the discovered articles to ascertain their precise adherence to the inclusion criteria. Subsequently, they affirmed whether the full text satisfied the inclusion criteria. If any inconsistencies emerged, a third reviewer was responsible for resolving them. The two reviewers collected relevant study data using a predetermined table for data collection. The researchers gathered pertinent information regarding the study, including the author’s identity, study design, publication year, sample size, duration, and patient group characteristics. In addition, the researchers gathered patient attributes, such as gender, age, initial measurements, ethnicity, and outcomes.

#### 2.1.2 Eligibility criteria

The present study examines the inclusion and exclusion criteria utilized in the research design (Soto et al., 2025). To identify suitable publications, the researchers employed specific inclusion and exclusion criteria. The inclusion criteria for this study consisted of the following:

1. The study population had to consist of patients diagnosed with type 2 diabetes mellitus (T2DM);
2. The interventions under investigation involved the comparison of at least two different GLP-1 receptor agonists, with one of the groups necessarily including the administration of Semaglutide, without concurrent metformin therapy. Moreover, we included interventions that studied the efficacy outcomes of switching from Dulaglutide or Liraglutide therapy to Semaglutide, without concurrent metformin therapy.
3. The efficacy outcomes being assessed were levels of HbA1c and fasting blood sugar (FBS); weight (kg), body mass index (BMI).
4. The study design had to be observational cohort studies (both retrospective and prospective), randomized controlled trials, or early-phase clinical trials published in peer-reviewed journals accompanied by complete analysis.

The exclusion criteria encompassed the following:

1. The intervention of interest was the administration of GLP1 receptor agonists in conjunction with other antidiabetic agents.
2. Studies assessing outcomes that were deemed irrelevant were excluded.
3. Only appropriately designed clinical trials were considered, excluding studies with evident biases or other study designs.

#### 2.1.3 Study selection

Two authors (MG and FM) independently assessed the included trials using the Cochrane Risk of Bias Tool (Sterne et al., 2019), which is specifically designed for randomized controlled trials. The Newcastle-Ottawa Scale was employed to evaluate observational studies (Wells et al., 2000), while the methodological quality of each included study was appraised using the modified Jadad scale (Jadad et al., 1996). The modified Jadad Scale was used in this meta-analysis due to its ease of application, reliability, and ability to assess key methodological elements such as randomization, blinding, and withdrawals, which are crucial for minimizing bias in RCTs. Its widespread acceptance and empirical validation further support its role in ensuring the inclusion of high-quality studies. In cases of disagreement between the two authors during study evaluation and selection, consensus was reached through consultation with a third reviewer (MK).

### 2.2 Quality assessment and risk of bias

The revised Jadad scale comprises eight components for evaluation purposes. These components include: whether randomization was conducted (with a score range of 0–1), whether the randomization process was appropriate (with a score range of −1 to 1), whether blinding was implemented (with a score range of 0–1); whether the blinding process was appropriate (with a score range of −1 to 1); whether the study provided information on withdrawals and dropouts (with a score range of 0–1); whether the study described the inclusion and exclusion criteria (with a score range of 0–1); whether adverse reactions were assessed (with a score range of 0–1); and whether the study described the statistical analysis (with a score range of 0–1). The quality of each study is assessed on a scale ranging from 0 (indicating the lowest quality) to eight (indicating the highest quality). The Cochrane risk of bias tool assessed seven categories: random-sequence generation, allocation concealment, blinding of outcome assessment, blinding of participants and personnel, selective reporting, completeness of data, and additional biases. To assess bias, each domain was categorized into one of three risk levels: low, unclear, or high. (Supplementary Material) The study quality was assessed using the Newcastle-Ottawa Scale, which consists of eight domains: representativeness, selection, baseline result, exposure ascertainment, comparability, follow-up duration, outcome assessment, and adequacy of follow-up.

### 2.3 Data management

The statistical analyses were conducted using R version 4.2.1 (R studio). The study presented dichotomous variables in numerical form, specifically as proportions. Continuous variables, on the other hand, were displayed as either mean ± standard deviation (SD) or median with interquartile range (IQR). Risk ratios (RRs) with 95% confidence intervals (CI) were computed using the random-effects model to analyze a dichotomous outcome. To assess the variations in continuous variables before and during the administration of Semaglutide and Tirzepatide, we utilized standard mean differences (SMDs) as a measure of comparison. The variability among the research included in the analysis was assessed using Cochran’s Q test and Higgins’s I2 values. The *I* ^2^ values of 25%, 50%, and 75% indicated low, moderate, and high levels of heterogeneity, respectively. A subgroup analysis was performed to examine the presence of heterogeneity. Publication bias was assessed using sensitivity analysis and Begg’s test. The statistical significance level was established at a significance level of P < 0.05.

### 2.4 Data analysis

When comparing GLP-1 agonists like Liraglutide and Semaglutide, meta-analyses were conducted using statistical software for data science, specifically Stata 17. A fixed-effect model with a 95% confidence interval (95% CI) was employed for continuous data using inverse variance when no statistical heterogeneity was observed (p > 0.05 in the ChI2 statistics) (DerSimonian and Laird, 1986). Otherwise, a random-effect model with a 95% CI was used for continuous data with Hedges’ (adjusted) g model and restricted maximum likelihood method (DerSimonian and Laird, 1986). Hedges’ g adjusts the effect size calculations for small samples with fewer than 20 participants, unlike Cohen’s d (Lakens, 2013). The effect size interpretation followed the common criteria for both Hedges’ g (Hedges and Olkin, 2014) and Cohen’s d (Cohen, 2013): small (0.2), medium (0.5), or large (0.8) (Brydges, 2019). The standard deviations of the mean changes from baseline were considered missing outcome data (Jpt, 2008), hindering the meta-analysis without missing SDs, as reported by previous systematic reviews (Shida et al., 2021; Yagiz et al., 2021). To calculate the missing SDs, the following formula was used (Jpt, 2008; Borenstein, 2009):

$$\text{SD change}=\sqrt{{SD}^{2\;}baseline+{SD}^{2}final-\left(2\times r\times SD\;baseline\times SDfinal\right)}$$

Where *SD change* is the SD of the mean changes from baseline, *SD baseline* is the SD of the pre-test, *SD final* is the SD of the post-test, and *r* is the correlation between the baseline and final measurements. This correlation value was usually not reported in the studies. For example, none of the studies eligible for this systematic review presented this *R*-value. Therefore, the *SD change* value was computed by assigning a value of 0.7 to *r* in formula (41), which provided a conservative estimate (Rosenthal, 1986) from previous systematic reviews (Papadopoulos et al., 2020; McGirr et al., 2015). If the included articles did not report SD, SD was calculated using the reported confidence interval or standard errors *via* the RevMan calculator. If the included articles did not report the SD final, the SD final was assumed to be the same as the *SD baseline*. Cochran’s *Q* test and the *I*-squared statistic were used (Page et al., 2021b)to assess the heterogeneity. *I* ^2^ values of less than 25%, 50%, and more than 75% indicated low, moderate, and high heterogeneity, respectively (with caution). Significant heterogeneity (*I* ^2^ > 75%, p < 0.05) suggested potential moderating effects among the included papers. Meta-regression was performed to conduct the moderator analysis. Potential moderators were total mean age, gender (the number of male participants), duration of treatment, and dose of prescribed GLP1 agonists. Moreover, funnel plots and Egger’s linear regression test were employed to evaluate publication bias (Hupe, 2019). The trim and fill method was applied to adjust the results in case of possible publication bias (Ouzzani et al., 2016). Lastly, the jackknife method (the “leave-one-out method”) was used for sensitivity analysis.

In the case of switching from Dulaglutide or Liraglutide to Semaglutide, meta-analysis was conducted using the Review Manager (RevMan) version 5.4.1 (The Cochrane Collaboration, 2020). We used a significance level of 0.05 for all analyses. We calculated mean differences (MD) with 95% confidence intervals (CI) for continuous variables. We derived standard deviations (SD) from the sample size, standard error (SE), or 95% CI when studies did not report SD. We measured heterogeneity using Cochrane’s Q and *I* ^2^ statistics. We considered heterogeneity to be high if p < 0.10 and *I* ^2^ > 50%. We chose a fixed-effects model when heterogeneity was low; otherwise, we opted for a random-effects model. We also examined the impact of each study on the direction of the pooled effect size by removing it from the analysis.

## 3 Results

### 3.1 Study selection

A comprehensive systematic search yielded a total of 1,570 records from PubMed (n = 215), Scopus (n = 906), Web of Science (n = 220), and Cochrane Library (n = 229). After removing 240 duplicate entries, 1,330 unique records remained for screening. During the initial screening of titles and abstracts, 894 articles were excluded due to irrelevance to the study objectives. The remaining 436 records were reviewed for full-text retrieval, of which 412 could not be accessed or were not suitable for full-text review. A total of 24 full-text articles were then assessed for eligibility based on predefined inclusion criteria. Of these, 8 studies were excluded due to a lack of appropriate pairing or the absence of a diabetic group. Ultimately, 16 studies fulfilled the eligibility criteria and were included in both the qualitative and quantitative analyses. The entire selection process is illustrated in the PRISMA flow diagram (Figure 1).

### 3.2 Semaglutide vs dulaglutide

#### 3.2.1 Study characteristics

In this section, four studies were thoroughly examined, all of which were RCT studies. The combined study population consisted of 660 individuals in the Dulaglutide group and 662 individuals in the Semaglutide group. Among these participants, males comprised 729 individuals, comprising 55.1% of the total population. The average age across all cohorts was 57.4 years, from 55.5 to 62.6 years. The duration of follow-up varied among the studies, ranging from 12 to 40 months, with an average duration of 29 months. The average dosage of Dulaglutide utilized in these studies ranged from 0.75 mg weekly to 1.5 mg weekly, with an average dose of 1.125 mg weekly. Regarding Semaglutide, the mean dosage administered during these studies varied from 0.5 mg to 1 mg weekly, with an average of 0.81 mg weekly.

#### 3.2.2 HbA1c changes

**FIGURE 2:**
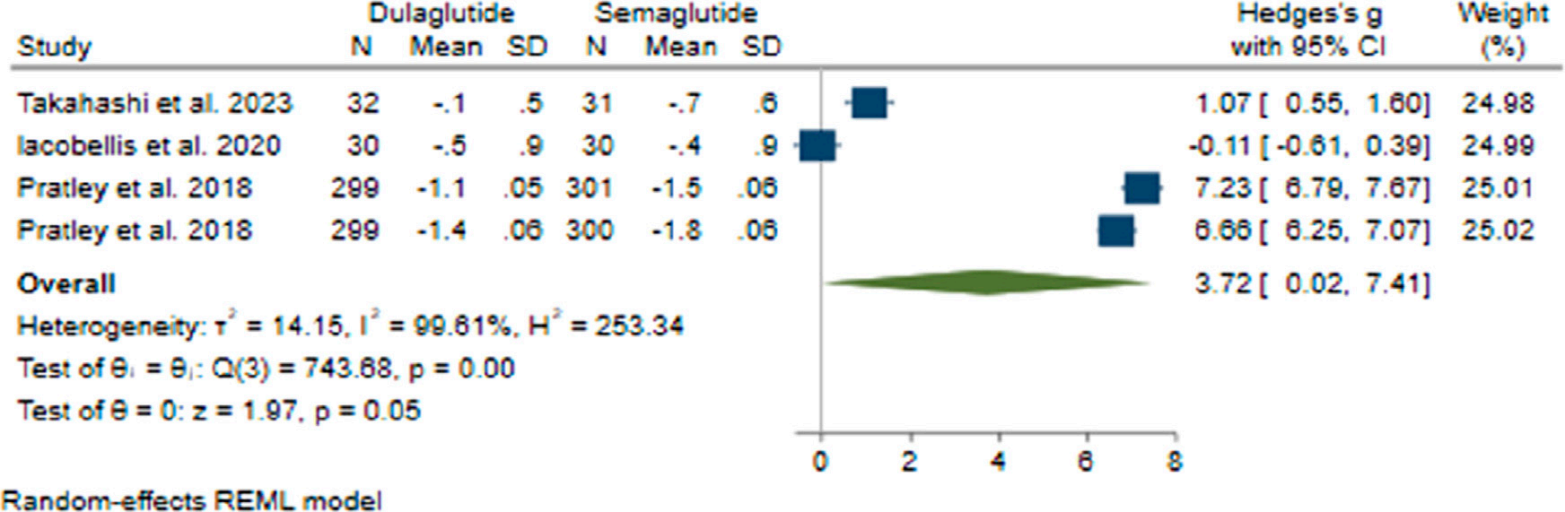
The impact of Semaglutide *versus* Dulaglutide on HbA1c changes.

Evaluating the impact of Semaglutide compared to Dulaglutide on HbA1c alteration, our analysis uncovered a notable advantage of Semaglutide in reducing HbA1c levels when compared with Dulaglutide. The calculated pooled effect size of 3.72 (95% CI: 0.02–7.41; p = 0.05) indicates a large difference in efficacy between the two medications (Figure 2). Sensitivity analysis, removing individual studies one by one, showed variations in the pooled effect size of HbA1c changes, ranging from 2.54 (95% CI: −1.55–6.64; p = 0.22) to 4.99 (95% CI: 1.14–8.84; p = 0.01). The study by Pratley et al. (2018) considerably influenced the pooled effect size, with its removal altering the effect size to 2.73 (95% CI: −1.73–7.20; p = 0.023).

Noteworthy heterogeneity was observed among the studies (*H* ^2^ = 253.34, *I* ^2^ = 99.61%, p < 0.001). Univariate meta-regression analyses revealed significant associations between the total number of male participants (b = 0.021; 95% CI: 0.017–0.026; p < 0.001; *R* ^2^ = 97.15%) and treatment duration (b = 0.26; 95% CI: 0.17–0.36; p < 0.001; *R* ^2^ = 91.54%) with HbA1c changes. The funnel plot displayed a symmetrical distribution, suggesting no publication bias, supported by Begg’s test (p = 0.73), although Egger’s test indicated a significant small study effect (p = 0.002). No imputed studies were found using the trim and fill model.

#### 3.2.3 Weight changes

**FIGURE 3:**
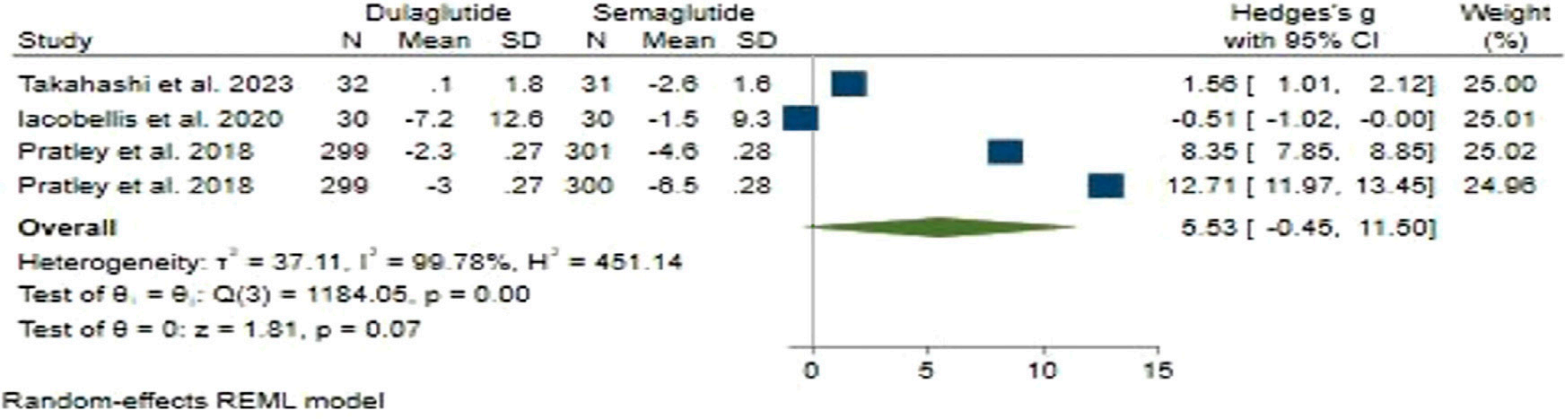
The impact of Semaglutide *versus* Dulaglutide on Weight changes.

Regarding weight change, Semaglutide did not demonstrate an advantage over Dulaglutide. In our analysis, the pooled effect size was determined to be 5.53 (95% CI: −0.45–11.5; p = 0.07), indicating a large level of disparity between the two treatments (Figure 3). Sensitivity analysis revealed variations in the pooled effect size of weight changes, ranging from 3.14 (95% CI: −2.11–8.38; p = 0.24) to 7.54 (95% CI: 1.19–13.89; p = 0.02), with the study by Iacobellis and Villasante Fricke (2020) significantly impacting the pooled effect size.

Substantial heterogeneity was observed among the studies (*H* ^2^ = 451.14, *I* ^2^ = 99.78%, p < 0.001). Univariate meta-regression analyses showed significant associations of the total number of male participants (*b* = 0.033; 95% CI: 0.018–0.049; p < 0.001; *R* ^2^ = 85.28%) and treatment duration (*b* = 0.417; 95% CI: 0.194–0.640; p < 0.001; *R* ^2^ = 80.81%) with weight changes. The funnel plot showed a symmetrical distribution, indicating no publication bias, supported by Egger’s test (p = 0.16) and Begg’s test (p = 0.73), with no imputed studies found using the trim and fill model.

#### 3.2.4 FBS changes

**FIGURE 4:**
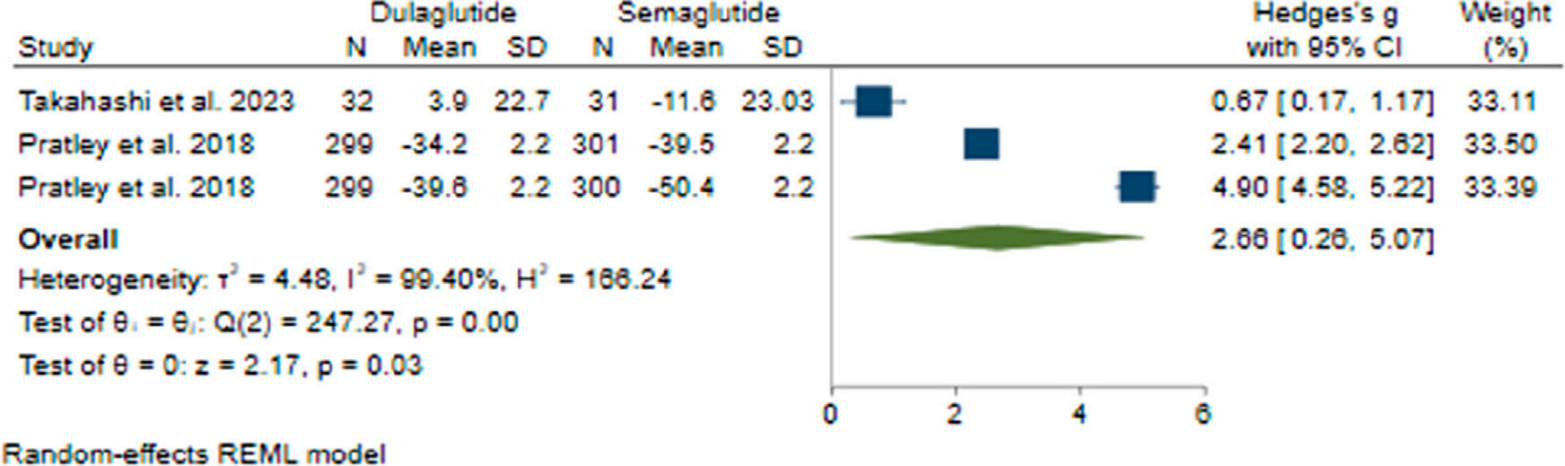
The impact of Semaglutide and Dulaglutide on FBS levels.

In comparing the effects of Semaglutide and Dulaglutide on FBS change, Semaglutide exhibited significantly greater efficacy than Dulaglutide. Based on a pooled effect size of 2.66 (95% CI: 0.26–5.07; p = 0.03), there appears to be a large difference between the two therapeutic agents (Figure 4). Sensitivity analysis revealed the significant impact of the study by Prately et al. (Pratley et al., 2018) on the pooled effect size.

Significant heterogeneity was found among the studies (*H* ^2^ = 166.24, *I* ^2^ = 99.40%, p < 0.001). Univariate meta-regression analysis showed a noteworthy association of the dose of administered weekly Dulaglutide with FBS changes (*b* = 4.46; 95% CI: 0.54–8.38; p = 0.026; *R* ^2^ = 67.18%). The funnel plot exhibited a symmetrical distribution, indicating no publication bias, supported by Egger’s test (p = 0.53) and Begg’s test (p = 1.0), with no imputed studies found using the trim and fill model.

#### 3.2.5 BMI changes

**FIGURE 5:**
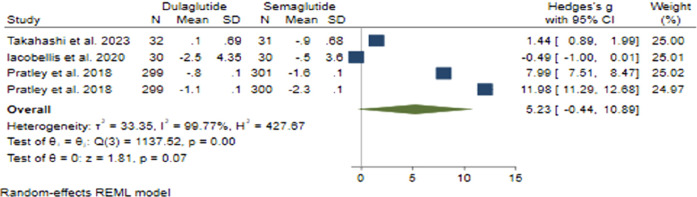
The effect of Semaglutide and Dulaglutide on BMI alterations.

Regarding BMI change with a pooled effect size of 5.23 (95% CI: −0.44–10.89; p = 0.07), Semaglutide did not demonstrate superiority over Dulaglutide (Figure 5). Sensitivity analysis revealed variations in the pooled effect size of BMI changes, ranging from 4.31 (95% CI: −3.29–11.90; p = 0.266) to 7.14 (95% CI: 1.12–13.16; p = 0.20), with the study by Prately et al. (Pratley et al., 2018) meaningfully impacting the pooled effect size.

Considerable heterogeneity was noted across the research studies (*H* ^2^ = 427.67, *I* ^2^ = 99.77%, p < 0.001). Univariate meta-regression analyses indicated noteworthy correlations between the total count of male participants (b = 0.032; 95% CI: 0.017–0.046; p < 0.001; *R* ^2^ = 86.17%) and treatment duration (*b* = 0.396; 95% CI: 0.188–0.604; p < 0.001; *R* ^2^ = 81.45%) with BMI changes. The funnel plot displayed a symmetrical distribution, suggesting the absence of publication bias, a finding reinforced by Egger’s test (p = 0.73) and Begg’s test (p = 0.26), and no additional studies were identified through the trim and fill model.

### 3.3 Semaglutide vs liraglutide

#### 3.3.1 Study characteristics

In this part, a total of eight studies were examined, of which seven were Randomized Controlled Trials (RCTs), and one was a cohort study. The study population comprised 1,248 individuals in the Liraglutide group and 2,104 individuals in the Semaglutide group, with males accounting for 1,594 individuals, constituting 47.5% of the total population. The mean age across all cohorts was 56.55 years, ranging from 47 to 69.5 years. The follow-up duration varied among the studies, ranging from 12 months to 68 months, with an average duration of 36 months. The mean dosage of Liraglutide in these studies ranged from 0.78 mg to 3 mg daily, with an average dose of 1.75 mg daily. As for Semaglutide, the mean dosage administered during these studies varied from 0.65 mg (fixed weekly injectable doses of 0.5 or 1 mg) to 98 mg weekly (14 mg daily oral doses), with an average of 13.3 mg weekly.

#### 3.3.2 HbA1c changes

**FIGURE 6:**
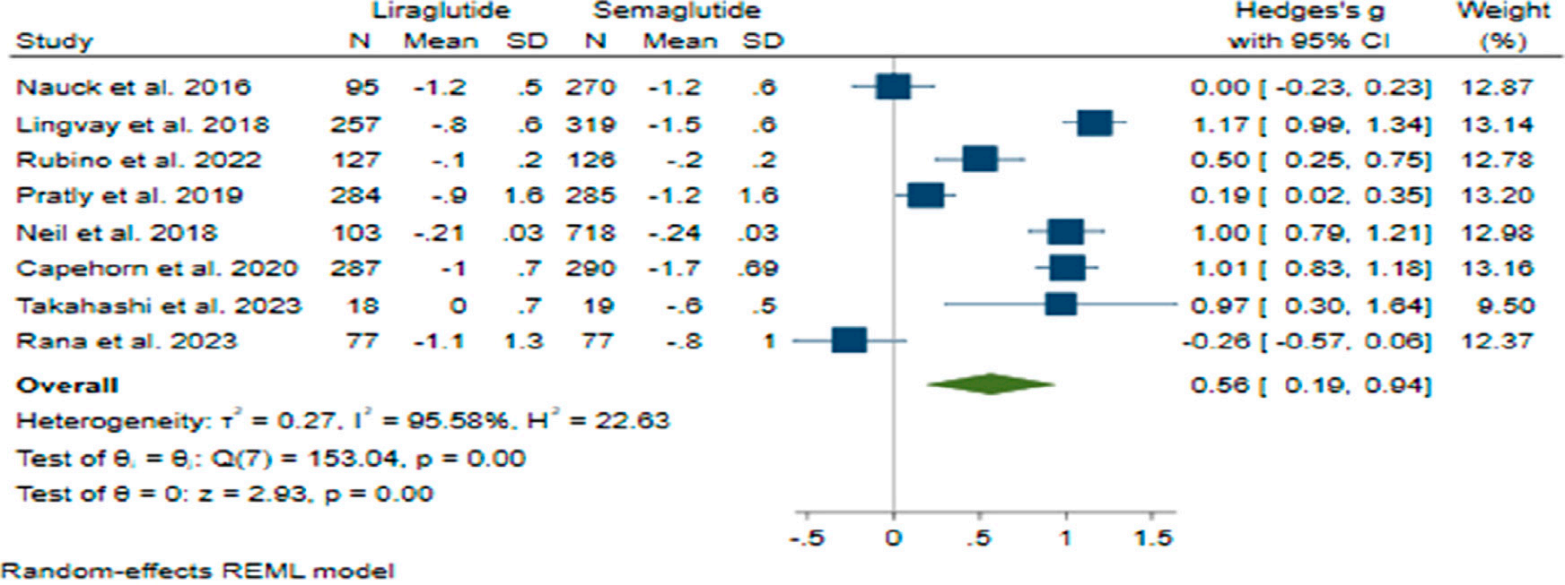
Comparison of HbA1c changes between Semaglutide and Liraglutide.

The comparison between Semaglutide and Liraglutide regarding their impact on HbA1c levels yielded a pooled effect size of 0.56 (95% CI: 0.19–0.94; p < 0.001), indicating a significant superiority of Semaglutide over Liraglutide in reducing HbA1c with a large difference in efficacy between the two drugs (Figure 6). Sensitivity analysis, excluding individual studies, revealed varying effect sizes ranging from 0.47 to 0.68, suggesting that no single study significantly influenced the overall estimate. Despite substantial heterogeneity observed among the studies (*H* ^2^ = 22.63, *I* ^2^ = 95.58%, p < 0.001), the source of this heterogeneity remained unidentified through meta-regression analysis.

The symmetrical distribution observed in the funnel plot’s visual inspection and non-significant results from Egger’s test (p = 0.91) and Begg’s test (p = 0.71) indicated the absence of publication bias. Furthermore, the trim and fill model did not identify any missing studies, corroborating the robustness of the findings.

#### 3.3.3 Weight changes

**FIGURE 7:**
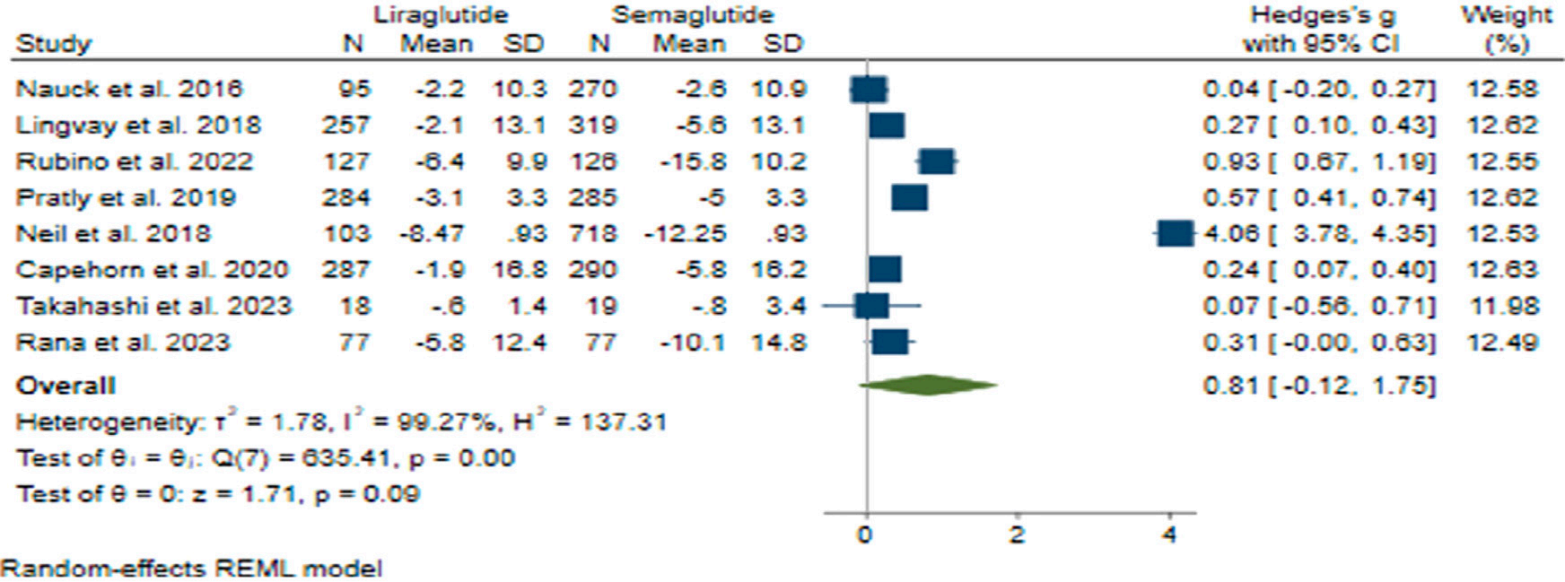
The impact of Semaglutide *versus* Liraglutide on weight changes.

The pooled effect size for weight reduction was calculated at 0.81 (95% CI: −0.12–1.75; p = 0.09), suggesting a lack of substantial difference between the efficacy of Semaglutide and Liraglutide (Figure 7). Upon conducting sensitivity analysis and excluding individual studies, there was observed variance in effect sizes, notably influenced by the study conducted by Neil et al. (O'Neil et al., 2018). Considerable heterogeneity was noted among the studies (*H* ^2^ = 137.31, *I* ^2^ = 99.27%, p < 0.001), and meta-regression analysis indicated a correlation between the daily dosage of Liraglutide and alterations in weight.

Although the funnel plot displayed an asymmetrical distribution, examinations for small-study effects did not provide evidence supporting publication bias. Three additional studies were incorporated on the right side of the funnel plot through the utilization of the trim and fill model, resulting in an adjustment of the collective effect size.

#### 3.3.4 FBS change

**FIGURE 8:**
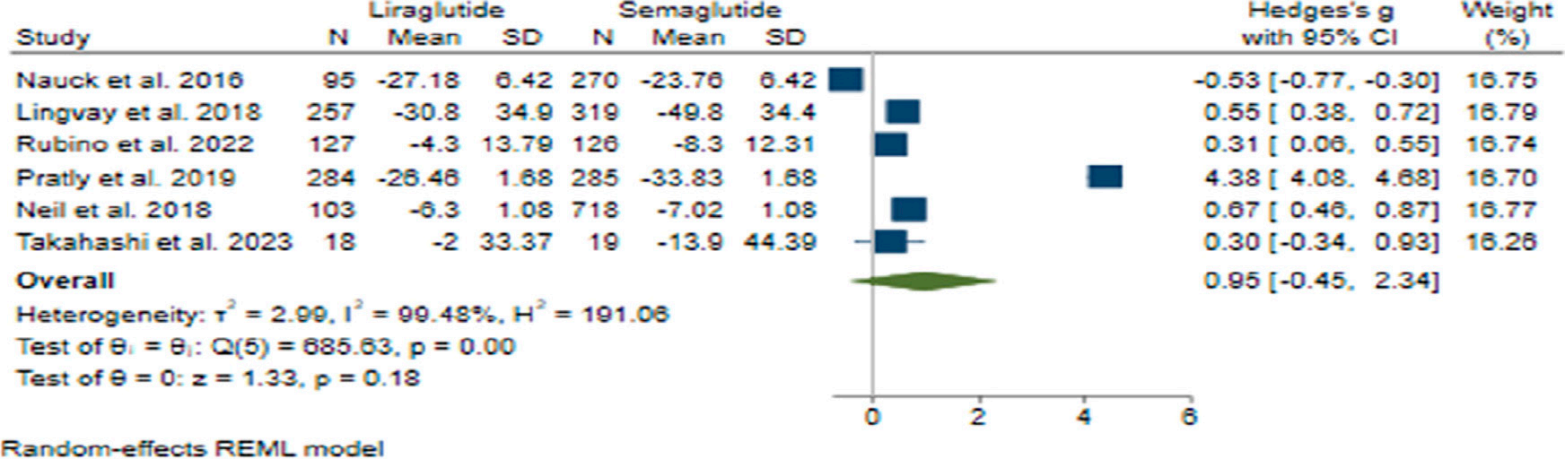
The effect of Semaglutide and Liraglutide on FBS.

In the analysis of FBS change, the combined effect size was calculated to be 0.95 (95% CI: −0.45–2.34; p = 0.18), suggesting no significant difference in efficacy between Semaglutide and Liraglutide (Figure 8). Upon conducting sensitivity analysis and excluding individual studies, varying effect sizes were observed, with none exerting a substantial influence on the overall estimation. Notably, considerable heterogeneity was detected among the studies (*H* ^2^ = 191.06, *I* ^2^ = 99.48%, p < 0.001), and meta-regression analysis identified a correlation between the weekly dosage of Semaglutide and changes in FBS levels.

Furthermore, the funnel plot exhibited a symmetrical distribution, indicating the absence of publication bias. Statistical tests supported this observation, and the trim and fill model did not identify any missing studies, further validating the robustness of the findings regarding FBS change.

#### 3.3.5 BMI change

**FIGURE 9:**
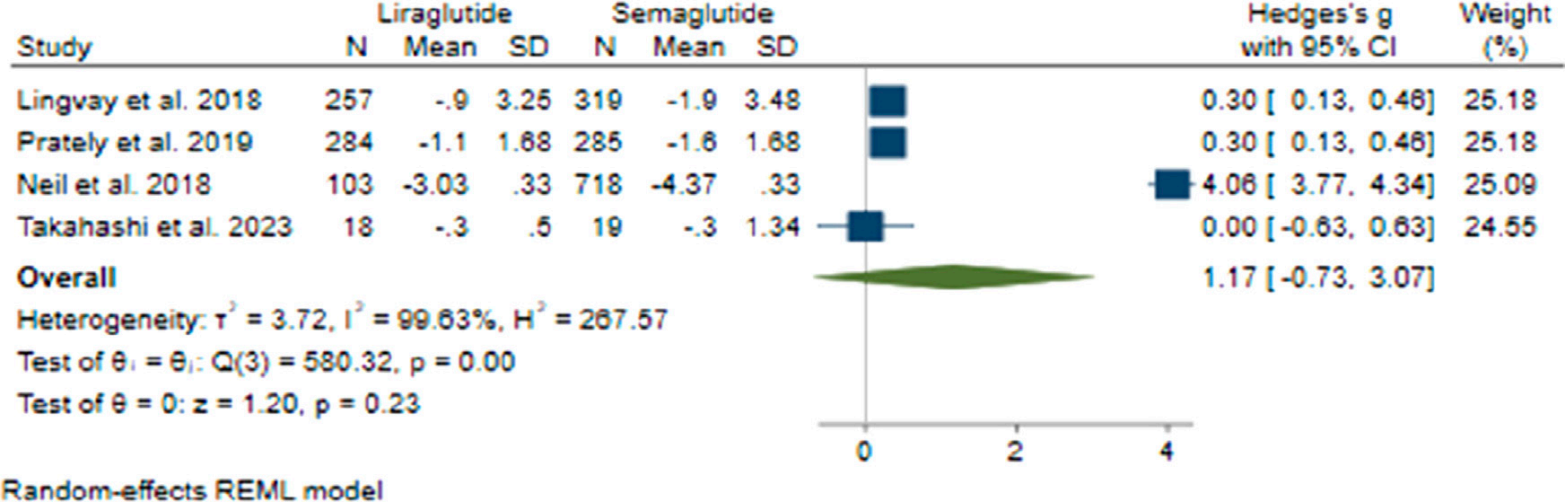
The effect of Semaglutide and Liraglutide on BMI.

In terms of changes in BMI, the collective effect size was found to be 1.17 (95% CI: −0.73–3.07; p = 0.23), indicating that there was no notable advantage of Semaglutide over Liraglutide (Figure 9). Upon conducting sensitivity analysis and excluding individual studies, it was noted that there were variations in effect sizes, with the study conducted by O’Neil et al. (2018) exerting a significant influence on the aggregated effect size. Notably, there was considerable heterogeneity among the studies (*H* ^2^ = 267.57, *I* ^2^ = 99.63%, p < 0.001), with meta-regression analysis revealing correlations between the daily dosage of Liraglutide, the average age of participants, and changes in BMI.

### 3.4 Tirzepatide vs semaglutide

#### 3.4.1 Study characteristics

This section undertook a comprehensive examination of two RCT studies. The collective study cohort encompassed 1,028 participants assigned to the Tirzepatide group and 515 participants allocated to the Semaglutide group. Of these individuals, males constituted 504 participants, constituting 65.4% of the total cohort. The mean age across both cohorts stood at 59.3 years, with an age range from 56.3 to 62.3 years. The follow-up duration ranged from 28 to 40 months, with an average duration of 34 months. The dosage of Tirzepatide employed in both of these studies was 15 mg weekly. Semaglutide was administered at 1 mg weekly in these two studies.

#### 3.4.2 HbA1c changes

**FIGURE 10:**
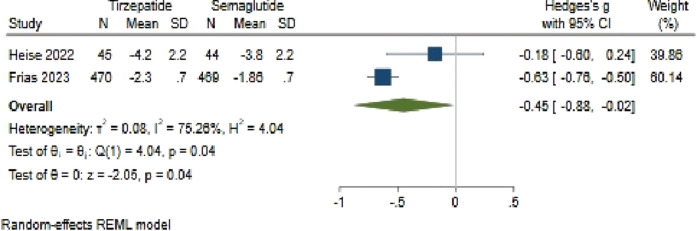
Impact of Semaglutide and Tirzepatide on HbA1c changes.

The analysis comparing HbA1c change between Tirzepatide and Semaglutide revealed a pooled effect size of −0.45 (95% CI: −0.88 to −0.02; p = 0.04), indicating a notable advantage of Tirzepatide over Semaglutide in reducing HbA1c levels, with a large difference in efficacy between the two medications (Figure 10). Importantly, the studies had substantial heterogeneity (*H* ^2^ = 4.11, *I* ^2^ = 75.66%, p < 0.001).

Upon investigating publication bias, an asymmetric distribution in the funnel plot suggested potential bias. To address this, one study was attributed to the right side of the funnel plot using a linear trimming estimator through the trim and fill model. Following this adjustment, the filled random-effects meta-analysis revealed an adjusted effect size of 0.62 (95% CI: 0.16–1.09).

#### 3.4.3 Weight changes

**FIGURE 11:**
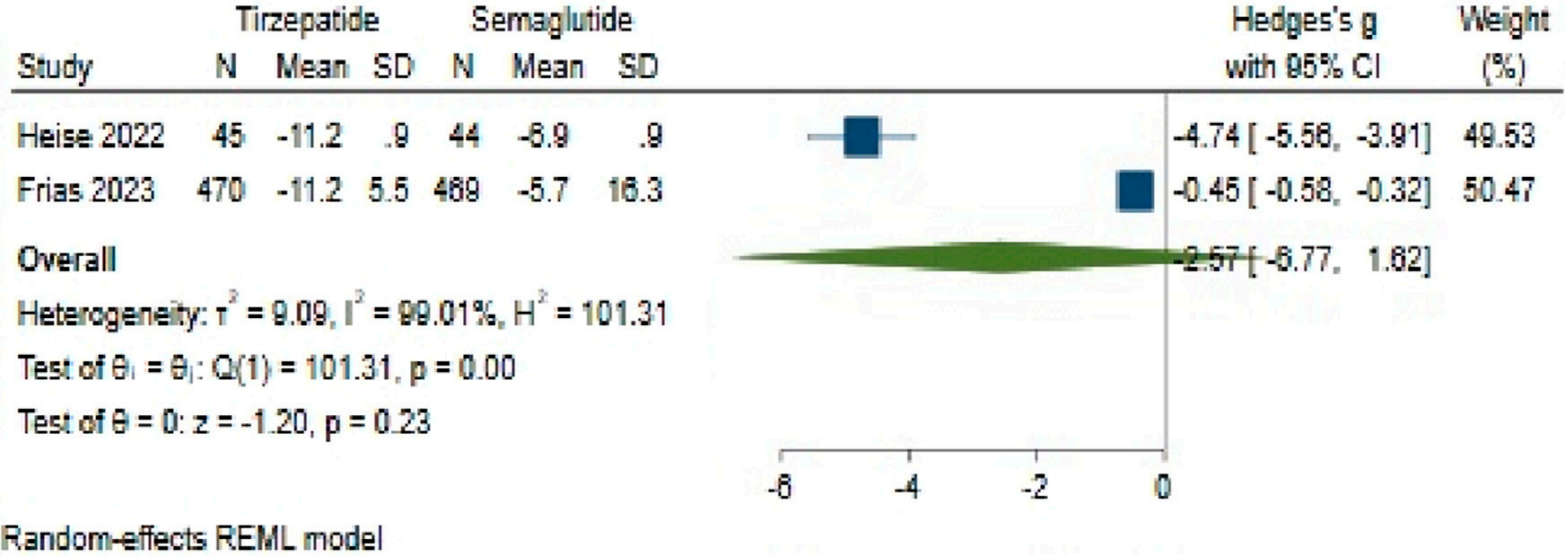
Effect of Semaglutide and Tirzepatide on weight changes.

Concerning weight change, Tirzepatide did not demonstrate clear superiority over Semaglutide, with a pooled effect size of −2.57 (95% CI: −6.77–1.62; p = 0.23) (Figure 11). Also, there was significant heterogeneity among the studies (*H* ^2^ = 105.15, *I* ^2^ = 99.05%, p < 0.001).

The analysis of publication bias, conducted *via* the utilization of a funnel plot, demonstrated an asymmetrical distribution. Using a linear trimming estimator in the trim and fill model, one study was credited on the right side of the funnel plot. Post-adjustment, the filled random-effects meta-analysis calculated an effect size of 0.45 (95% CI: −4.38–5.28).

#### 3.4.4 FBS changes

**FIGURE 12:**
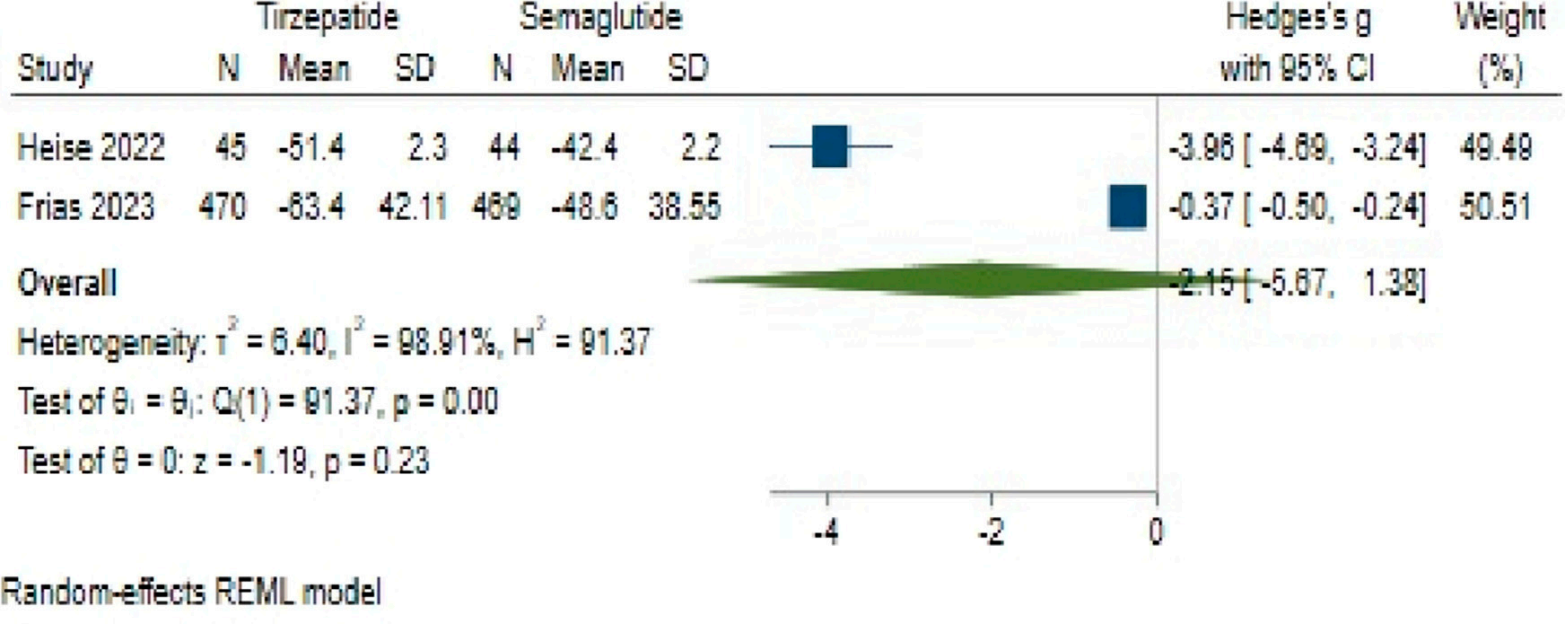
Impact of semaglutide and tirzepatide on FBS alterations.

Regarding FBS change, Tirzepatide did not demonstrate a clear advantage over Semaglutide, with a pooled effect size of −2.15 (95% CI: −5.67–1.38; p = 0.18) (Figure 12). Likewise, substantial heterogeneity persisted among the studies (*H* ^2^ = 94.62, *I* ^2^ = 99.94%, p < 0.001).

An assessment of publication bias also indicated an asymmetric distribution through the funnel plot. Using a linear trimming estimator in the trim and fill model, one study was attributed to the left side of the funnel plot. Following this adjustment, the filled random-effects meta-analysis yielded an effect size of 0.36 (95% CI: −3.69–4.42).

### 3.5 Switching from liraglutide to semaglutide

#### 3.5.1 Study characteristics

We comprehensively analyzed four studies focusing on transitioning from Liraglutide to Semaglutide. Of these, two were RCTs, while the remaining two were cohort studies. The aggregate study population encompassed 226 individuals. Among these participants, males accounted for 129 individuals, constituting 57.1% of the total population. The mean age across all cohorts was 56.7 years, ranging from 48 to 61.5 years. The duration of follow-up varied across the studies, spanning from 12 to 48 months, with an average follow-up duration of 27.5 months. In two of these four studies, the Semaglutide dosages employed were 0.5 mg and 0.75 mg weekly.

#### 3.5.2 HbA1c changes

**FIGURE 13:**
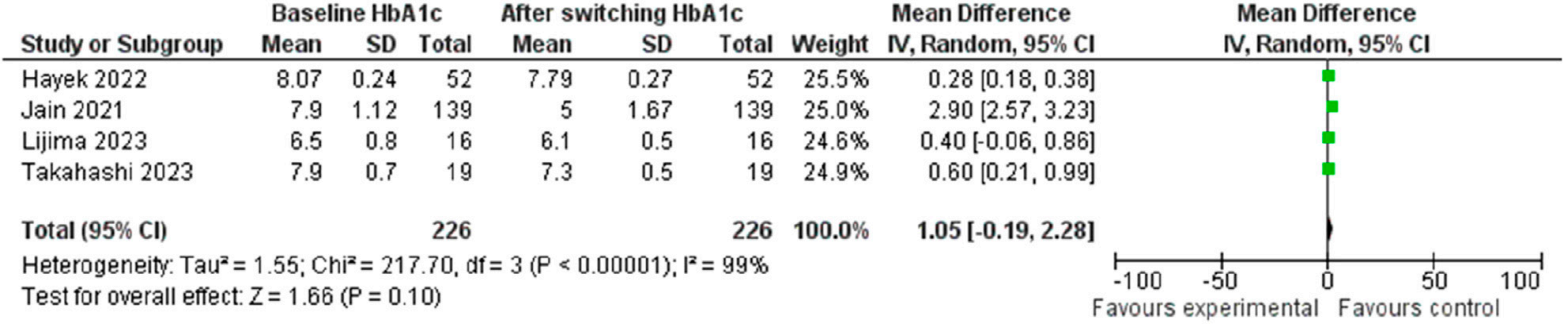
Effect of transitioning treatment from Liraglutide to Semaglutide on HbA1c alterations.

The investigation into HbA1c change following the transition from Liraglutide to Semaglutide revealed a pooled effect size of 1.05 (95% CI: −0.19–2.28; p = 0.10), indicating that switching medications did not result in a significant reduction in HbA1c levels (Figure 13). Sensitivity analysis demonstrated variations in the pooled effect size of HbA1c changes, ranging from 1.30 (95% CI: −0.37–2.98; p = 0.13) to 0.35 (95% CI: 0.18–0.52; p < 0.001) upon the exclusion of individual studies, with the study by Jain et al. (2021) significantly influences the result.

Notably, there was substantial heterogeneity among the studies (*I* ^2^ = 99.61%, p < 0.001), although this decreased significantly after the removal of the Jain et al. (Jain et al., 2021) study (*I* ^2^ = 0.24, p < 0.001). Analysis of publication bias indicated a symmetrical distribution in the funnel plot, suggesting the absence of publication bias.

#### 3.5.3 Weight changes

**FIGURE 14:**
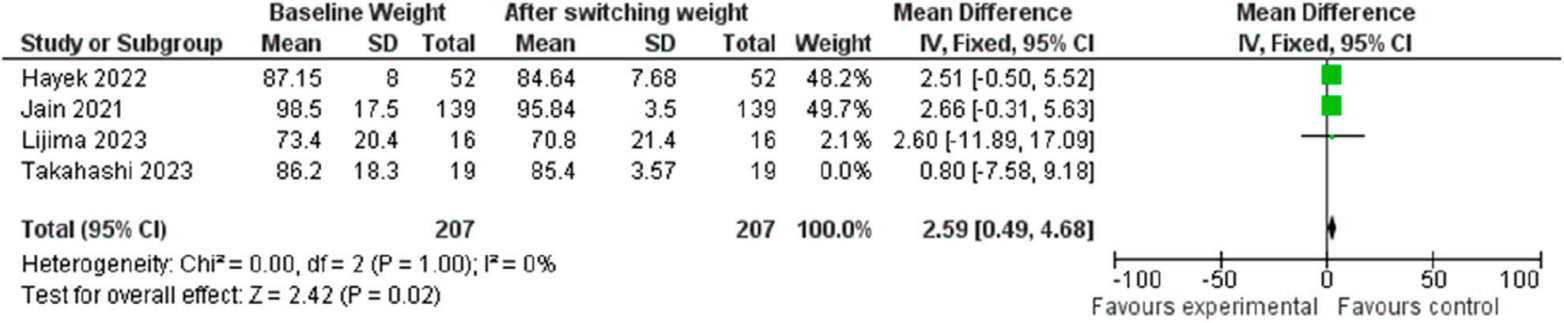
Impact of switching treatment from Liraglutide to Semaglutide on weight changes.

In terms of weight change, transitioning from Liraglutide to Semaglutide was associated with a pooled effect size of 2.48 (95% CI: 0.45–4.51; p = 0.02), signifying a significant weight reduction (Figure 14). Sensitivity analysis displayed variations in the pooled effect size of weight changes, ranging from 2.32 (95% CI: −0.46–5.11; p = 0.10) to 2.59 (95% CI: 0.49–4.68; p = 0.02) upon excluding individual studies, with the Jain et al. (McGirr et al., 2015) study exerting a notable impact. Heterogeneity was absent among the studies (*I* ^2^ = 0, p = 0.98), prompting the utilization of a fixed-effect model for result reporting.

Similarly, the examination of publication bias revealed a symmetrical distribution in the funnel plot, indicating no publication bias.

#### 3.5.4 FBS change

**FIGURE 15:**
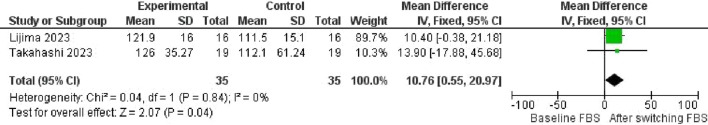
Impact of switching treatment from Liraglutide to Semaglutide on FBS changes.

Regarding FBS change, the transition from Liraglutide to Semaglutide was accompanied by a pooled effect size of 10.76 (95% CI: 0.55–20; p = 0.04), indicating a significant reduction in FBS levels (Figure 15). No heterogeneity was observed among the studies (*I* ^2^ = 0%, p = 0.84), leading to using a fixed-effect model for result reporting.

Additionally, examination of publication bias indicated a symmetrical distribution in the funnel plot, suggesting the absence of publication bias.

### 3.6 Switching from dulaglutide to semaglutide

#### 3.6.1 Study characteristics

In this part of our investigation, we thoroughly examined three studies centered on the transition from Dulaglutide to Semaglutide. Among these studies, one constituted an RCT, while the other was a cohort study. The collective study cohort comprised 69 individuals. Within this cohort, males constituted 44 individuals, representing 64% of the population. The mean age across all study cohorts was 58.8 years, ranging from 54 to 64.6 years. The mean duration of follow-up varied among the studies, ranging from 6 to 48 months, with an average follow-up period of 39 months. The Semaglutide dosages utilized in these three studies were 0.75 mg and 1 mg weekly.

#### 3.6.2 HbA1c changes

**FIGURE 16:**
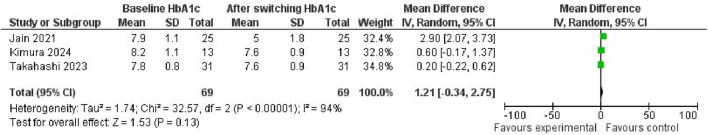
Effect of transitioning treatment from Dulaglutide to Semaglutide on HbA1c changes.

The investigation into the change in HbA1c levels consequent to the transition from Dulaglutide to Semaglutide yielded a pooled effect size of 1.21 (95% CI: −0.34, 2.75; p = 0.13), indicating a lack of significant alteration in HbA1c (Figure 16). Noteworthy heterogeneity was evident among the studies (*I* ^2^ = 94%, p < 0.001), underscoring substantial variability in outcomes across the analyzed datasets.

Upon visual scrutiny of the funnel plot, a symmetrical distribution emerged, suggesting the absence of publication bias in the examined data.

#### 3.6.3 Weight changes

**FIGURE 17:**
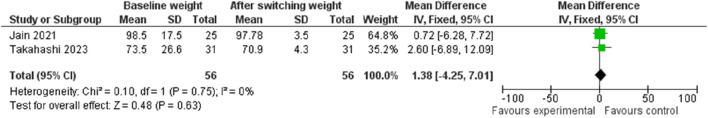
Effect of transitioning treatment from dulaglutide to semaglutide on weight changes.

Concerning weight change, transitioning from Dulaglutide to Semaglutide resulted in a pooled effect size of 1.38 (95% CI: −4.25, 7.01; p = 0.63), indicative of no substantial impact on weight (Figure 17). Notably, no discernible heterogeneity was observed among the studies (*I* ^2^ = 0%, p = 0.75), prompting the utilization of a fixed-effect model for result reporting.

Moreover, visual inspection of the funnel plot unveiled a symmetrical distribution, thus indicating the absence of publication bias within the analyzed dataset.

## 4 Discussion

Our systematic review and meta-analysis aimed to explore how Semaglutide compares to three other GLP-1 receptor agonists (GLP-1 RAs): Dulaglutide, Liraglutide, and Tirzepatide. Additionally, we assessed the effects of transitioning from Dulaglutide or Liraglutide to Semaglutide. Our primary focus was on examining the impact of reducing HbA1c levels, FBS levels, BMI, and changes in weight among patients with T2DM. Our findings offer valuable insights into the relative efficacy of these GLP-1 RAs in clinical practice.

Various meta-analyses have examined the efficacy of GLP-1 receptor agonists in the management of type 2 diabetes mellitus; however, the majority have included placebo-controlled trials or studies incorporating combination therapies. While these analyses provide valuable insights into the general effectiveness of GLP-1 receptor agonists, they do not allow for a direct comparative assessment between different agents within this drug class. Such methodological limitations introduce potential confounding factors, particularly when additional glucose-lowering therapies, such as insulin or metformin, are co-administered.

In contrast, the present study focuses exclusively on RCTs that directly compare at least two GLP-1 receptor agonists as monotherapies. By restricting the analysis to studies where neither treatment arm involved placebo or adjunctive therapies, we aimed to eliminate potential biases arising from heterogeneity in background treatments. This approach enhances the precision of our findings by ensuring that any observed differences in clinical outcomes—such as HbA1c reduction, fasting plasma glucose levels, weight loss, and BMI—are attributable solely to the intrinsic efficacy of the GLP-1 receptor agonists being compared. Consequently, this meta-analysis provides a more rigorous and clinically relevant evaluation of the relative therapeutic benefits of Semaglutide, Liraglutide, Dulaglutide, and Tirzepatide, facilitating a more evidence-based approach to treatment selection in the management of type 2 diabetes and obesity.

### 4.1 Glycemic control

Our analysis indicates that Semaglutide is more effective than Dulaglutide in managing T2DM. Semaglutide, administered once weekly at dosages ranging from 0.5 to 1 mg, showed superior efficacy compared to Dulaglutide, dosed once weekly from 0.75 to 1.5 mg, reducing both HbA1c and FBS levels. Similarly, Semaglutide, administered once weekly at dosages ranging from 0.65 to 0.98 mg, demonstrated significantly better efficacy than Liraglutide, which ranges from 0.78 to 3 mg daily, in reducing HbA1c levels. However, both medications showed comparable efficacy in terms of FBS change. Furthermore, Tirzepatide, administered once weekly at 15 mg, proved to be more effective than Semaglutide, administered once weekly at 1 mg, in reducing HbA1c levels. However, there was no apparent advantage in FBS alteration. Our study revealed no significant difference in HbA1c levels when switching from Dulaglutide to Semaglutide, administered once weekly at 0.75 and 1 mg. Similarly, switching from Liraglutide to Semaglutide, given once weekly at 0.5 and 0.75 mg, showed no significant difference in HbA1c levels. However, there was a noticeable reduction in FBS levels after the switch.

Li et al. (2018) conducted a meta-analysis to compare the effectiveness of Semaglutide with that of placebo and other active comparators, including Dulaglutide. Their study found that Semaglutide significantly decreased HbA1c and FBS levels by 0.85% compared to the other comparators. In 2019, Mishriky et al. (2019) conducted a study comparing the effectiveness of Liraglutide once-daily (QD) with other GLP-1 RA and dipeptidyl peptidase-4 (DPP-4) inhibitors across five 12-week clinical trials. The study found that Semaglutide 1 mg was significantly more effective in reducing HbA1c levels than other treatments. Our research findings align with these results. Our research supports the findings of Lingvay et al. (2022a) that Semaglutide is a more effective treatment than Dulaglutide in lowering HbA1c levels. While Lingvay et al. observed significant reductions in both HbA1c and body weight with a 2.0 mg dose of Semaglutide in comparison to doses of 3.0 and 4.5 mg of Dulaglutide, our study showed that doses of Semaglutide ranging from 0.5 to 1 mg were superior to doses of Dulaglutide administered from 0.75 to 1.5 mg in reducing HbA1c levels.

A study conducted by Alsugair et al. (2021) found that Semaglutide 1 mg QW is more effective in reducing HbA1c levels than Liraglutide 1.2 and 1.8 mg. The study also revealed that Semaglutide 0.5 mg QW is more effective than Liraglutide 1.2 mg. Our study findings align with Karagiannis et al. (2024) and Müllertz et al. (2024), who also concluded that Tirzepatide 15 mg has a more significant impact on HbA1c levels than subcutaneous Semaglutide. The SUSTAIN 7 trial (O'Neil et al., 2018) discovered that individuals with T2DM experienced better clinical outcomes and reduced direct costs when treated with once-weekly Semaglutide at doses of 0.5 and 1 mg compared to Dulaglutide 1.5 mg. Improvements in HbA1c levels primarily drove the long-term benefits. Although the cost of treatment with once-weekly Semaglutide was higher than that of Dulaglutide, Liraglutide, and Tirzepatide, the improved outcomes resulted in increased survival, which offset the initial expenditure. While there were modest per-patient cost savings, the higher dose of once-weekly Semaglutide led to statistically significant savings. The cost-effectiveness of the treatment may be influenced by adherence, which is impacted by treatment goals and tolerability (Witkowski et al., 2018; Palmer et al., 2004; Viljoen et al., 2019).

### 4.2 Body weight loss

Patients with T2DM treated with certain GLP-1RAs have reported experiencing weight loss (Vilsbøll et al., 2012). Concerning weight change and BMI reduction, our analysis found no significant difference between Dulaglutide (administered at dosages ranging from 0.75 to 1.5 mg once weekly) and Semaglutide (administered at dosages ranging from 0.5 to 1 mg once weekly). Similarly, Liraglutide (administered at dosages ranging from 0.78 to 3 mg daily) and Semaglutide (administered at dosages ranging from 0.65 to 98 mg once weekly) exhibited comparable efficacy in terms of weight reduction and BMI.

It was found that there was no significant difference in weight modification between the use of Tirzepatide at a dosage of 15 mg once weekly and Semaglutide at a dosage of 1 mg once a week. However, when transitioning from Dulaglutide to Semaglutide, administered at dosages of 0.75 mg and 1 mg once a week, respectively, there was no significant difference in weight changes. Interestingly, when switching from Liraglutide to Semaglutide, administered at 0.5 and 0.75 mg once a week, respectively, there was a noticeable decrease in weight.

Semaglutide and Liraglutide share similar structural features but exhibit distinct differences. Semaglutide has a larger linker molecule and an extended fatty acid derivative than Liraglutide. Furthermore, there is a notable positional alteration at residue eight, where Ala in Liraglutide is replaced by Amino-isobutyric acid in Semaglutide. This modification enhances albumin binding and reinforces stability against DPP-4 degradation (Nauck et al., 2012; Lund et al., 2014). Research suggests that Semaglutide’s unique mechanism of action in managing weight and its molecular dimensions may explain its superiority over Liraglutide in weight reduction (Lund et al., 2014; Madsbad, 2016). However, Semaglutide is more potent than other incretin-based therapies. However, it is associated with an increased risk of gastrointestinal side effects such as nausea, vomiting, and diarrhea, which often leads to treatment discontinuation. Semaglutide may offer advantages over other incretin-based therapies if patients can tolerate the gastrointestinal adverse effects and are willing to use an injectable medication. For patients who have a history of gastrointestinal intolerance, an alternative incretin-based therapy may be preferred over Semaglutide (Nathan and Group, 2014; Control et al., 1993; Nauck, 2016). According to the research conducted by Lingvay et al. (2022b), Semaglutide 2.0 mg significantly reduced body weight compared to Dulaglutide 3.0 and 4.5 mg. However, after our investigation, we found no clear advantage in weight loss with Semaglutide (administered at doses ranging from 0.5 to 1 mg) compared to Dulaglutide (administered at doses ranging from 0.75 to 1.5 mg).

In a study conducted by Alsugair et al. (2021), it was found that Semaglutide is more effective in reducing weight than Liraglutide 0.6 mg. The study concluded that Semaglutide 0.5 and 1 mg, administered once weekly, are significantly more effective in weight reduction than Liraglutide 0.6 mg. However, no significant difference was found between Semaglutide and Liraglutide 1.2 and 1.8 mg. In contrast, our study found that Semaglutide (administered at dosages ranging from 0.65 to 98 mg) and Liraglutide (administered at dosages ranging from 0.78 to 3 mg) exhibited similar effectiveness in weight loss. Additionally, our study revealed that Tirzepatide 15 mg did not present any significant advantage in weight modification compared to Semaglutide. This finding contradicts the results of prior studies conducted by Karagiannis et al. (2024) and Müllertz et al. (2024), who observed that Tirzepatide 15 mg had a more significant impact on weight reduction compared to subcutaneous Semaglutide. However, our research yielded different results, as we observed no noticeable advantage in weight modification.

As mentioned in our results part, the ratio of male participants could be correlated with HbA1c changes. Consistently, several recent studies have been conducted to assess the possible sex-dependent effect of GLP-1Ras. These differences are thought to stem from variations in hormonal regulation and receptor expression that influence both metabolic responses and drug tolerability. These findings highlight the importance of including sex as a factor when optimizing GLP-1–based treatment strategies (Börchers and Skibicka, 2024; Rentzeperi et al., 2022).

Our research study faced limitations primarily due to the high heterogeneity among included studies and the relatively low number of articles in our meta-analysis. For example, only two articles compared Semaglutide vs Tirzepatide. To address this, we attempted to identify potential sources of heterogeneity by conducting meta-regression analyses on variables such as the average drug dosage, study duration, average age, and proportion of male participants. However, despite these efforts, some studies still exhibited unexplained heterogeneity, introducing a potential source of bias into our findings. To enhance the accuracy and reliability of our study, it is imperative to include more studies in future analyses to ensure more comprehensive and unbiased results. Moreover, we noted the influence of publication bias in some studies, suggesting a potential bias in the journals’ selection of articles for publication. To mitigate this issue, more studies should be published over time to ensure a more representative sample of research findings. Finally, most of the included trials recruited patients from the USA, Europe, and some Asian countries, which limits the generalizability of the results across all ethnic groups.

Future research endeavors should prioritize identifying the variables responsible for heterogeneity in meta-regression analyses, including factors such as gender, drug dosage, and study duration, which can impact changes in HbA1c and weight. While our meta-analysis utilized average drug dosages, further investigations are needed to determine the equivalent doses of Liraglutide to Semaglutide. These insights hold significant implications for clinicians and policymakers tasked with managing T2DM effectively.

## 5 Conclusion

Semaglutide exhibited greater efficacy in reducing HbA1c levels than Dulaglutide and Liraglutide. Conversely, Tirzepatide demonstrated superior effectiveness over Semaglutide. However, these medications did not confer a significant weight change or BMI reduction advantage. Switching from Dulaglutide or Liraglutide to Semaglutide did not lead to a significant impact on HbA1c levels. Nevertheless, it did contribute to weight reduction and lowered FBS levels. Further investigation is warranted to determine the optimal dosages and long-term effects of medications for T2DM and identify the variables contributing to heterogeneity through meta-regression analysis, mostly due to differences in study design, patient demographics, and treatment regimes.

## Data Availability

The datasets presented in the study can be found in online repositories. The names of the repository/repositories and accession number(s) can be found below: https://osf.io/u2axy/.
