# Supplementary material for: Comparative effectiveness of semaglutide versus liraglutide, dulaglutide or tirzepatide: a systematic review and meta-analysis
Source: Front Pharmacol. 2025 May 15;16:1438318. doi: 10.3389/fphar.2025.1438318 (PMC12120964; doi:10.3389/fphar.2025.1438318)
Supplement: Supplementary file 1 [file Supplementaryfile1.docx]

**Supplementary Table 1. Search Strategies for PubMed, Scopus, Web of Science, and Cochrane**

| **Search Engine** | **Search strategy** | **Additional**  **filters** | **No.** |
| --- | --- | --- | --- |
| **PubMed** | (semaglutide[Supplementary Concept]) AND (((tirzepatide[Supplementary Concept]) OR (Liraglutide[MeSH Terms])) OR (dulaglutide[Supplementary Concept])) | **English,**  **5 February 2025** | 215 |
| **Scopus** | ( ( TITLE-ABS-KEY ( tirzepatide ) OR TITLE-ABS-KEY ( mounjaro ) OR TITLE-ABS-KEY ( ly3298176 ) OR TITLE-ABS-KEY (liraglutide) OR TITLE-ABS-KEY (Victoza) OR TITLE-ABS-KEY (Saxenda) OR TITLE-ABS-KEY (NN 2211) OR TITLE-ABS-KEY (2211, NN) OR TITLE-ABS-KEY (NN2211) OR TITLE-ABS-KEY (NN-2211) OR TITLE-ABS-KEY ( dulaglutide ) OR TITLE-ABS-KEY ( LY 2189265 ) OR TITLE-ABS-KEY ( LY-2189265 ) OR TITLE-ABS-KEY ( LY2189265 ) OR TITLE-ABS-KEY (Trulicity) ) ) AND ( ( TITLE-ABS-KEY ( semaglutide ) OR TITLE-ABS-KEY ( rybelsus ) OR TITLE-ABS-KEY ( ozempic ) ) ) | **English,**  **5 February 2025** | 906 |
| **Web Of Science** | #1: Tirzepatide (Title) or Mounjaro (Title) or LY3298176 (Title)  #2: liraglutide (Title) or Victoza (Title) or Saxenda (Title) or NN 2211 (Title) or 2211, NN (Title) or NN2211 (Title) or NN-2211 (Title)  #3: dulaglutide (Title) or LY 2189265 (Title) or LY-2189265 (Title) or LY2189265 (Title) or Trulicity (Title)  #4: Semaglutide (Title) or rybelsus (Title) or Ozempic (Title)  #5: #1 OR #2 OR #3  Final: #4 And #5 | **English,**  **5 February 2025** | 220 |
| **Cochrane** | #1: (tirzepatide):ti,ab,kw OR (Mounjaro):ti,ab,kw OR (LY3298176):ti,ab,kw  #2: MeSH descriptor: [Liraglutide] explode all trees  #3: (dulaglutide):ti,ab,kw OR (LY 2189265):ti,ab,kw OR (LY-2189265):ti,ab,kw OR (LY2189265):ti,ab,kw OR (Trulicity):ti,ab,kw  #4: (semaglutide):ti,ab,kw OR (Rybelsus):ti,ab,kw OR (Ozempic):ti,ab,kw  #5: #1 OR #2 OR #3  Final: #4 And #5 | **English,**  **5 February 2025** | 229 |

**Supplementary Table 2. Assessment of Risk of Bias**

| **Study** | **Random sequence generation** | **Allocation concealment** | **Selective reporting** | **Blinding of participants / personal** | **Blinding of outcome assessment** | **Incomplete outcome data** | **Other bias** |
| --- | --- | --- | --- | --- | --- | --- | --- |
| Heise et al.(1) | 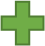 | 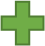 | 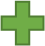 | 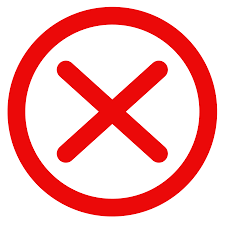 | 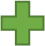 | 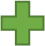 | 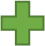 |
| Frias et al.(2) | 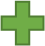 | 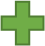 | 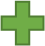 | 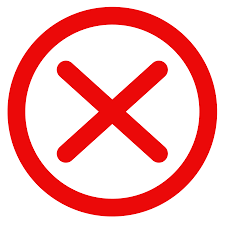 | 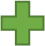 | 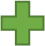 | 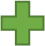 |
| Takahashi et al.(3) | 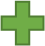 | 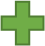 | 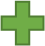 | 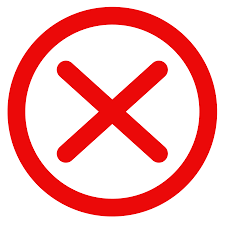 | 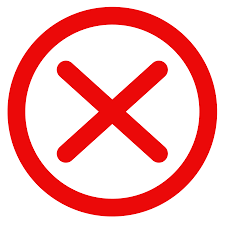 | 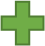 | 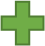 |
| Pratley RE et al. (4) | 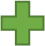 | 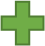 | 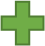 | 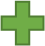 | 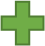 | 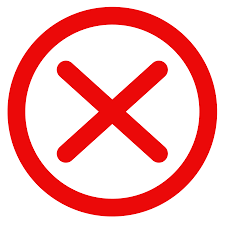 | 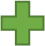 |
| T.Iijima et al.(5) | 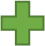 | 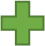 | 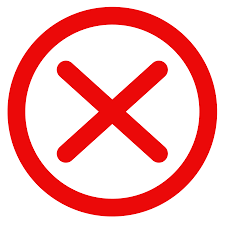 | 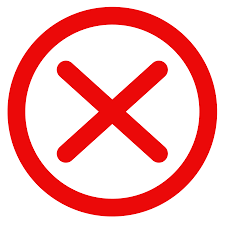 | 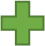 | 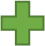 | 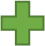 |
| Pratley et al.(6) | 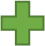 | 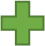 |  | 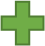 | 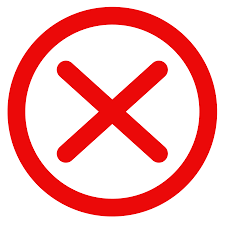 | 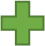 | 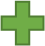 |
| O’Neil et al.(7) | 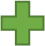 | 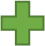 | 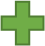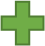 | 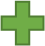 | 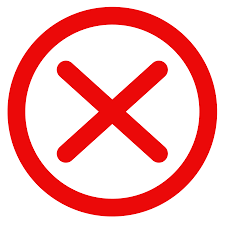 | 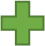 | 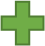 |
| Capehorn et al.(8) | 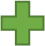 | 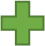 | 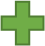 | 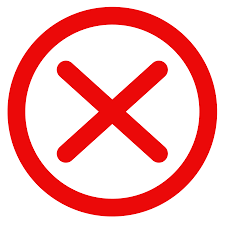 | 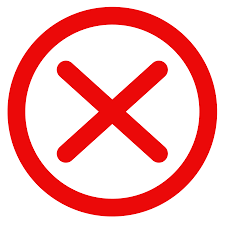 | 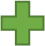 | 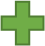 |
| Nauck et al.(9) | 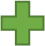 | 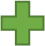 | 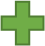 | 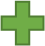 | 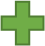 | 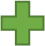 | 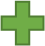 |
| Lingvay et al.(10) | 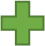 | 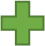 | 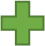 | 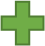 | 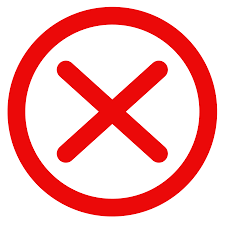 | 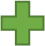 | 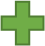 |
| Rubino et al.(11) | 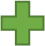 | 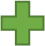 | 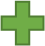 | 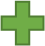 | 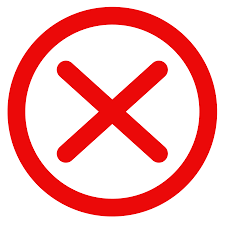 | 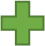 | 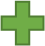 |

**Supplementary Table 3: Tirzepatide vs Semaglutide**

| **Author** | **Published year** | **Study design** | **Duration (Weeks)** | **Mean dose (mg) of weekly Semaglutide** | **Mean dose (mg) of weekly Tirzepatide** | **Population Semaglutide** | **Population Tirzepatide** | **Total mean age (SD)** | **Total male number (%)** | **Baseline HbA1c (%) Semaglutide (SD)** | **Baseline HbA1c (%) Tirzepatide (SD)** | **Baseline weight (kg) Semaglutide (SD)** | **Baseline weight(kg) Tirzepatide (SD)** | **Baseline FBS (mg/dl) Semaglutide (SD)** | **Baseline FBS (mg/dl) Tirzepatide (SD)** | **Baseline BMI (kg/m2) Semaglutide (SD)** | **Baseline BMI (kg/m2) Tirzepatide (SD)** |
| --- | --- | --- | --- | --- | --- | --- | --- | --- | --- | --- | --- | --- | --- | --- | --- | --- | --- |
| Heise et al.(1) | 2022 | RCT | 28 | 1 | 15 | 44 | 45 | 62.3 (6.5) | 65 (73%) | 7.70 (0.60) | 7.83 (0.72) | 92.65 (14.01) | 94.15 (13.99) | 128.6 (25.0) | 139.3 (30.2) | 30.82 (3.84) | 31.28 (5.01) |
| Frias et al. (2) | 2023 | RCT | 40 | 1 | 15 | 469 | 470 | 56.3 (10.6) | 439 (46%) | 8.25 (1.01) | 8.26 (1.00) | 93.7 (21.12) | 93.8 (21.83) | 171.4 (49.77) | 172.4 (54.37) | 34.2 (7.15) | 34.5 (7.11) |

**Supplementary Table 4: Liraglutide vs Semaglutide**

| **Author** | **Published year** | **Study design** | **Duration (weeks)** | **Mean dose (mg) of daily Liraglutide** | **Mean dose (mg) of weekly Semaglutide** | **Population Liraglutide** | **Population Semaglutide** | **Total mean age (SD)** | **Total male number (%)** | **Baseline HbA1c (%) Liraglutide (SD)** | **Baseline HbA1c (%) Semaglutide (SD)** | **Baseline weight (kg) Liraglutide (SD)** | **Baseline weight (kg) Semaglutide (SD)** | **Baseline FBS (mg/dl) Liraglutide (SD)** | **Baseline FBS (mg/dl) Semaglutide (SD)** | **Baseline BMI (kg/m2) Liraglutide (SD)** | **Baseline BMI (kg/m2) Semaglutide (SD)** |
| --- | --- | --- | --- | --- | --- | --- | --- | --- | --- | --- | --- | --- | --- | --- | --- | --- | --- |
| Nauck et al.(9) | 2016 | RCT | 12 | 1.5 | 0.65 | 95 | 270 | 55 (9.7) | 237 (64%) | 8.05 (0.74) | 8.11 (0.84) | 88.76 (13.29) | 86.51 (14.18) | 165 (38.69) | 170.19 (41.38) | 30.94 (4.6) | 30.72 (4.41) |
| Lingvay et al.(10) | 2018 | RCT | 26 | 0.78 | 7.91 | 257 | 319 | 56.7(9.9) | 310 (53%) | 8.1 (0.84) | 8.01 (0.8) | 93.77 (17.93) | 94.81 (18.85) | 170.05 (45.18) | 169.07 (44.53) | 32.76 (4.53) | 32.82 (4.17) |
| Rubino et al.(11) | 2022 | RCT | 68 | 3 | 2.4 | 127 | 126 | 48.5(13.6) | 54 (21%) | 5.5 (0.3) | 5.5 (0.3) | 103.7 (22.5) | 102.5 (25.3) | 95.2 (8.5) | 96.1 (10.2) | 37.2 (6.4) | 37.0 (7.4) |
| Pratley et al.(6) | 2019 | RCT | 52 | 1.8 | 98 (14 mg daily oral doses) | 284 | 285 | 56(10) | 296 (52%) | 8.0 (0.7) | 8.0 (0.7) | 95.5 (21.9) | 92.9 (20.6) | 167.56 (39.99) | 167.02 (40.18) | 33.4 (6.7) | 32.5 (5.9) |
| Neil et al.(7) | 2018 | RCT | 52 | 3 | 1.75 | 103 | 718 | 47(12) | 290 (35%) | 5·5 (0·4) | 5.48 (0.4) | 108·7 (21·9) | 111.35 (23.2) | 99.09 (237.6) | 98.07 (231.7) | 38·6 (6·6) | 39.28 (7.06) |
| Capehorn et al.(8) | 2020 | RCT | 30 | 1.2 | 1 | 287 | 290 | 59.5(10.3) | 237 (41%) | 8.3 (1.0) | 8.2 (0.9) | 97.2 (21.7) | 96.6 (21.0) | 178.37 (45.04) | 176.57 (41.44) | 33.7 (7.0) | 33.7 (6.6) |
| Takahashi et al.(3) | 2023 | RCT | 24 | 1.35 | 0.75 | 18 | 19 | 60.2(18.3) | 24 (64%) | 7.8 (1.0) | 7.9 (0.7) | 83.9 (21.3) | 86.2 (18.3) | 137.0 (35.2) | 126 (35.27) | 29.8 (3.4) | 30.1 (7.1) |
| Rana et al.(12) | 2023 | Cohort | 24 | 1.4 | 0.7 | 77 | 77 | 69.5(9.3) | 146 (94%) | 9.2 (1.7) | 8.9 (1.4) | 106.6 (20.3) | 108.6 (38.7) | **-** | **-** | **-** | **-** |

**Supplementary Table 5: Dulaglutide vs Semaglutide**

| **Author** | **Published year** | **Study design** | **Duration (weeks)** | **Mean dose (mg) of weekly Dulaglutide** | **Mean dose (mg) of weekly Semaglutide** | **Population Dulaglutide** | **Population Semaglutide** | **Total mean age (SD)** | **Total male number (%)** | **Baseline HbA1c (%) Dulaglutide (SD)** | **Baseline HbA1c (%) Semaglutide (SD)** | **Baseline weight (kg) Dulaglutide (SD)** | **Baseline weight (kg) Semaglutide (SD)** | **Baseline FBS (mg/dl) Dulaglutide (SD)** | **Baseline FBS (mg/dl) Semaglutide (SD)** | **Baseline BMI (kg/m2) Dulaglutide (SD)** | **Baseline BMI (kg/m2) Semaglutide (SD)** |
| --- | --- | --- | --- | --- | --- | --- | --- | --- | --- | --- | --- | --- | --- | --- | --- | --- | --- |
| Takahashi et al.(3) | 2023 | RCT | 24 | 0.75 | 0.75 | 32 | 31 | 62.6) 17( | 33 (52%) | 7.8 (1.0) | 7.8 (0.8) | 73.4 (15.3) | 73.5 (26.6) | 135.6 (28.9) | 142.8 (28.2) | 28.1 (4) | 26.9 (5) |
| lacobellis et al.(13) | 2020 | RCT | 12 | 1.5 | 1 | 30 | 30 | 56) 8.9( | 34 (56%) | 8.2 (1.2) | 7.3 (1.2) | 106 (21) | 105 (14) | - | - | 36.5 (6) | 34.3 (5) |
| Pratley et al.(4) | 2018 | RCT | 40 | 0.75 | 0.5 | 299 | 301 | 55.5)10.6( | 329 (54%) | 8.2 (0.9) | 8.3 (0.9) | 95.6 (23.0) | 96.4 (24.4) | 173.9 (47.7) | 176.3 (45.7) | 33.6 (6.9) | 33.7 (7.1) |
| Pratley et al.(4) | 2018 | RCT | 40 | 1.5 | 1 | 299 | 300 | 55.5)10.6( | 333 (55%) | 8.2 (0.9) | 8.2 (0.9) | 93.4 (21.8) | 95.5 (20.9) | 172.5 (41.2) | 177.1 (46.5) | 33.1 (6.6) | 33.6 (6.5) |

**Supplementary Table 6: Switching from Liraglutide to Semaglutide**

| **Author** | **Published year** | **Study design** | **Duration (weeks)** | **Mean dose (mg) of weekly Semaglutide** | **Population** | **Total mean age (SD)** | **Total male number (%)** | **Baseline HbA1c (%) Semaglutide (SD)** | **Baseline weight (kg) Semaglutide (SD)** | **Baseline FBS (mg/dl) Semaglutide (SD)** | **Baseline BMI (kg/m2) Semaglutide (SD)** |
| --- | --- | --- | --- | --- | --- | --- | --- | --- | --- | --- | --- |
| Jain et al.(14) | 2021 | Cohort | 48 | 1 | 139 | 58 (10.6) | 81 (58%) | 7.9 (1.1) | 98.5 (17.5) |  | 33.1 (5.63) |
| Takahashi et al.(3) | 2023 | RCT | 24 | 0.75 | 19 | 59.6 (18.4) | 12 (63%) | 7.9 (0.7) | 86.2 (18.3) | 126 (35.27) | 30.1 (7.1) |
| Hayek et al.(15) | 2022 | Cohort | 12 | 0.58 | 52 | 48 (6) | 25 (48%) | 8.2 (0.2) | 87.15 (8) | - | 32.5 (2.7) |
| Lijima et al.(5) | 2023 | RCT | 26 | 0.5 | 16 | 61.5 (11.2) | 11 (68%) | 6.5 (0.8) | 73.4 (20.4) | 121.9 (16) | 27 (6.6) |

**Supplementary Table 7: Switching from Dulaglutide to Semaglutide**

| **Author** | **Published**  **year** | **Study**  **design** | **Duration (weeks)** | **Mean dose (mg) of weekly Semaglutide** | **Population** | **Total mean age (SD)** | **Total male number (%)** | **Baseline HbA1c (%) Semaglutide (SD)** | **Baseline weight (kg) Semaglutide (SD)** | **Baseline FBS (mg/dl) Semaglutide (SD)** | **Baseline BMI (kg/m2) Semaglutide (SD)** |
| --- | --- | --- | --- | --- | --- | --- | --- | --- | --- | --- | --- |
| Jain et al.(14) | 2021 | Cohort | 48 | 1 | 25 | 58 (10.6) | 14 (56%) | 7.9 (1.1) | 98.5 (17.5) | - | 33.1 (5.63) |
| Takahashi et al.(3) | 2023 | RCT | 24 | 0.75 | 31 | 64.6 (16.3) | 20 (64%) | 7.8 (0.8) | 73.5 (26.6) | 142.8 (28.2) | 26.9 (5) |
| Kimura et al. (16) | 2024 | Cohort | 6 | 1 | 13 | 54 (9.9) | 10 (77%) | 8.2 (1.1) | 1.1 | - | - |

1. Heise T, Mari A, DeVries JH, Urva S, Li J, Pratt EJ, et al. Effects of subcutaneous tirzepatide versus placebo or semaglutide on pancreatic islet function and insulin sensitivity in adults with type 2 diabetes: a multicentre, randomised, double-blind, parallel-arm, phase 1 clinical trial. The Lancet Diabetes & Endocrinology. 2022;10(6):418-29.

2. Frías JP, Davies MJ, Rosenstock J, Pérez Manghi FC, Fernández Landó L, Bergman BK, et al. Tirzepatide versus semaglutide once weekly in patients with type 2 diabetes. New England Journal of Medicine. 2021;385(6):503-15.

3. Takahashi Y, Nomoto H, Yokoyama H, Takano Y, Nagai S, Tsuzuki A, et al. Improvement of glycaemic control and treatment satisfaction by switching from liraglutide or dulaglutide to subcutaneous semaglutide in patients with type 2 diabetes: A multicentre, prospective, randomized, open‐label, parallel‐group comparison study (SWITCH‐SEMA 1 study). Diabetes, Obesity and Metabolism. 2023.

4. Pratley RE, Aroda VR, Lingvay I, Lüdemann J, Andreassen C, Navarria A, et al. Semaglutide versus dulaglutide once weekly in patients with type 2 diabetes (SUSTAIN 7): a randomised, open-label, phase 3b trial. The lancet Diabetes & endocrinology. 2018;6(4):275-86.

5. Iijima T, Shibuya M, Ito Y, Terauchi Y. Effects of switching from liraglutide to semaglutide or dulaglutide in patients with type 2 diabetes: A randomized controlled trial. Journal of Diabetes Investigation. 2023;14(6):774-81.

6. Pratley R, Amod A, Hoff ST, Kadowaki T, Lingvay I, Nauck M, et al. Oral semaglutide versus subcutaneous liraglutide and placebo in type 2 diabetes (PIONEER 4): a randomised, double-blind, phase 3a trial. The Lancet. 2019;394(10192):39-50.

7. O'Neil PM, Birkenfeld AL, McGowan B, Mosenzon O, Pedersen SD, Wharton S, et al. Efficacy and safety of semaglutide compared with liraglutide and placebo for weight loss in patients with obesity: a randomised, double-blind, placebo and active controlled, dose-ranging, phase 2 trial. The Lancet. 2018;392(10148):637-49.

8. Capehorn M, Catarig A-M, Furberg J, Janez A, Price H, Tadayon S, et al. Efficacy and safety of once-weekly semaglutide 1.0 mg vs once-daily liraglutide 1.2 mg as add-on to 1–3 oral antidiabetic drugs in subjects with type 2 diabetes (SUSTAIN 10). Diabetes & metabolism. 2020;46(2):100-9.

9. Nauck MA, Petrie JR, Sesti G, Mannucci E, Courreges J-P, Lindegaard ML, et al. A phase 2, randomized, dose-finding study of the novel once-weekly human GLP-1 analog, semaglutide, compared with placebo and open-label liraglutide in patients with type 2 diabetes. Diabetes care. 2016;39(2):231-41.

10. Lingvay I, Desouza CV, Lalic KS, Rose L, Hansen T, Zacho J, et al. A 26-week randomized controlled trial of semaglutide once daily versus liraglutide and placebo in patients with type 2 diabetes suboptimally controlled on diet and exercise with or without metformin. Diabetes Care. 2018;41(9):1926-37.

11. Rubino DM, Greenway FL, Khalid U, O’Neil PM, Rosenstock J, Sørrig R, et al. Effect of weekly subcutaneous semaglutide vs daily liraglutide on body weight in adults with overweight or obesity without diabetes: the STEP 8 randomized clinical trial. Jama. 2022;327(2):138-50.

12. Rana KB, Britnell SR, Gilbertson ME, Ibrahim SL. Comparison of the effectiveness of Liraglutide vs Semaglutide in a veteran population. Journal of Pharmacy Practice. 2023;36(5):1095-101.

13. Iacobellis G, Villasante Fricke AC. Effects of semaglutide versus dulaglutide on epicardial fat thickness in subjects with type 2 diabetes and obesity. Journal of the Endocrine Society. 2020;4(4):bvz042.

14. Jain AB, Kanters S, Khurana R, Kissock J, Severin N, Stafford SG. Real-world effectiveness analysis of switching from liraglutide or dulaglutide to semaglutide in patients with type 2 diabetes mellitus: the retrospective REALISE-DM study. Diabetes Therapy. 2021;12:527-36.

15. Al Hayek AA, Al Dawish MA. Evaluation of patient-reported satisfaction and clinical efficacy of once-weekly semaglutide in patients with type 2 diabetes: an ambispective study. Advances in Therapy. 2022;39(4):1582-95.

16. Kimura T, Kubo M, Takahashi K, Wamata R, Iwamoto Y, Iwamoto H, et al. Usefulness of Once-Weekly GLP-1 Receptor Agonist Semaglutide on Glycemic Control in Subjects with Type 2 Diabetes Mellitus: Switching from the Same Class Dulaglutide in a Retrospective Observation Study. J Diabetes Res. 2024;2024:5880589.
